# Studying Conformational Properties of Transmembrane Domain of KCNE3 in a Lipid Bilayer Membrane Using Molecular Dynamics Simulations

**DOI:** 10.3390/membranes14020045

**Published:** 2024-02-04

**Authors:** Anna Clara Miranda Moura, Isaac K. Asare, Mateo Fernandez Cruz, Antonio Javier Franco Aguado, Kaeleigh Dyan Tuck, Conner C. Campbell, Matthew W. Scheyer, Ikponwmosa Obaseki, Steve Alston, Andrea N. Kravats, Charles R. Sanders, Gary A. Lorigan, Indra D. Sahu

**Affiliations:** 1 Natural Science Division, Campbellsville University, Campbellsville, KY 42718, USA; ammour424@students.campbellsville.edu (A.C.M.M.); mfern512@students.campbellsville.edu (M.F.C.); ajfran084@students.campbellsville.edu (A.J.F.A.); kdtuck070@students.campbellsville.edu (K.D.T.); salston@campbellsville.edu (S.A.); 2 Department of Chemistry and Biochemistry, Miami University, Oxford, OH 45056, USAkravatan@miamioh.edu (A.N.K.); lorigag@miamioh.edu (G.A.L.); 3 Department of Biochemistry and Center for Structural Biology, Vanderbilt University, Nashville, TN 37232, USA; chuck.sanders@vanderbilt.edu

**Keywords:** molecular dynamics simulation, KCNE3 transmembrane domain, conformational dynamics, lipid bilayers

## Abstract

KCNE3 is a single-pass integral membrane protein that regulates numerous voltage-gated potassium channel functions such as KCNQ1. Previous solution NMR studies suggested a moderate degree of curved α-helical structure in the transmembrane domain (TMD) of KCNE3 in lyso-myristoylphosphatidylcholine (LMPC) micelles and isotropic bicelles with the residues T71, S74 and G78 situated along the concave face of the curved helix. During the interaction of KCNE3 and KCNQ1, KCNE3 pushes its transmembrane domain against KCNQ1 to lock the voltage sensor in its depolarized conformation. A cryo-EM study of KCNE3 complexed with KCNQ1 in nanodiscs suggested a deviation of the KCNE3 structure from its independent structure in isotropic bicelles. Despite the biological significance of KCNE3 TMD, the conformational properties of KCNE3 are poorly understood. Here, all atom molecular dynamics (MD) simulations were utilized to investigate the conformational dynamics of the transmembrane domain of KCNE3 in a lipid bilayer containing a mixture of POPC and POPG lipids (3:1). Further, the effect of the interaction impairing mutations (V72A, I76A and F68A) on the conformational properties of the KCNE3 TMD in lipid bilayers was investigated. Our MD simulation results suggest that the KCNE3 TMD adopts a nearly linear α helical structural conformation in POPC-POPG lipid bilayers. Additionally, the results showed no significant change in the nearly linear α-helical conformation of KCNE3 TMD in the presence of interaction impairing mutations within the sampled time frame. The KCNE3 TMD is more stable with lower flexibility in comparison to the N-terminal and C-terminal of KCNE3 in lipid bilayers. The overall conformational flexibility of KCNE3 also varies in the presence of the interaction-impairing mutations. The MD simulation data further suggest that the membrane bilayer width is similar for wild-type KCNE3 and KCNE3 containing mutations. The Z-distance measurement data revealed that the TMD residue site A69 is close to the lipid bilayer center, and residue sites S57 and S82 are close to the surfaces of the lipid bilayer membrane for wild-type KCNE3 and KCNE3 containing interaction-impairing mutations. These results agree with earlier KCNE3 biophysical studies. The results of these MD simulations will provide complementary data to the experimental outcomes of KCNE3 to help understand its conformational dynamic properties in a more native lipid bilayer environment.

## 1. Introduction

KCNE3 is an integral membrane protein containing a single transmembrane domain that modulates the function of voltage-gated potassium channels including KCNQ1 [1,2,3,4]. Voltage-gated K^+^ channels are critical for the function of cardiac, nervous and auditory systems and represent promising targets for various therapeutic agents [5,6]. Previous structural studies of KCNE3 in isotropic bicelles suggested that the extracellular N-terminal helix associated with membrane surface containing amino acid residues 10 to 30 is connected with a flexible loop to the TMD [7]. The transmembrane domain assembles as an alpha-helical region (residues 57 to 82) embedded in the membrane and is important for its interaction with KCNQ1 during the channel gating. The TMD is further connected to a small juxtamembrane helix containing residues 90 to 95 and further extended to a disorder C-terminus containing residues 96 to 103 [7]. The end of the extracellular N-terminal transmembrane domain of KCNE3 associates with the end of the extracellular S1 segment of KCNQ1, while the end of the C-terminal cytosolic transmembrane domain of KCNE3 interacts with the intracellular C-terminal of the S4 segment in the KCNQ1 voltage-sensing domain residing at the membrane/cytosol interface [7].

While the overall structure of KCNE3 is known, the TMD helix has been reported to assume different conformations. A solution NMR study of KCNE3 in isotropic bicelles (DMPG/DHPC) suggested that the TMD helix assumes a moderate degree of curvature having a bending observed close to the end of the TMD C-terminal [7]. The concave face of the curvature lies along the residues T71, S74 and G78 [7]. In contrast, a recent cryo-EM study of KCNE3/KCNQ1 using a truncated form of KCNE3 (amino acids 53-95) in nanodiscs captured an extended form of the KCNE3 TMD [8]. This study further suggested that the KCNE3 tucks its TMD helix against KCNQ1 to stabilize the up conformation of the voltage sensor, locking it in its ‘‘open’’ configuration [8]. Furthermore, certain mutations in the TMD of KCNE3 affect the interaction with KCNQ1. For example, in a study where KCNE3 mutants F68A, V72A and I76A were co-expressed with wild-type KCNQ1, reduced ratios of fluorescence change (ΔF−160mV/ΔF60mV) values than those of the KCNE3-WT/KCNQ1 channel were observed. These results suggest that these mutations impair the interaction between KCNQ1 and KCNE3 and cause a shift in the equilibrium of the S4 segment to the down position [9]. It is important to understand the helical conformation of KCNE3 TMD in lipid bilayers to better understand KCNE3’s structure–function relationship and structural perturbations when interacting with KCNQ1 during channel gating.

Recently, we studied structural topology and dynamic properties of KCNE3 in different lipid bilayer membranes including POPC (1-palmitoyl-2-oleoyl- *sn*-glycero-3-phosphocholine)/POPG(1-palmitoyl-2-oleoyl-*sn*-glycero- 3-phospho- (1′-*rac*-glycerol) (sodium salt)), DMPC and POPC alone lipid bilayers using all-atom molecular dynamics simulations [10]. These results suggested that the KCNE3 TMD is less flexible and more stable when compared to the N- and C-termini of KCNE3 in these three membrane environments. Our recent circular dichroism (CD) spectroscopic data suggested that the secondary structural folding of KCNE3 is better in POPC/POPG lipid bilayers when compared to that in dodecyl phosphatidylcholine (DPC) detergent micelles [11]. Additionally, continuous wave electron paramagnetic resonance (CW-EPR) lineshape analysis data suggested a restricted motion of KCNE3 in lipid-bilayer vesicles in comparison to that in detergent micelles [11]. Together, these studies indicate that the structural dynamic studies of KCNE3 in a native-like membrane bilayer environment is required for fully understanding the structure–function relationship of KCNE3 [11]. A native-like membrane environment provides a physiological state of the protein. Despite the vast amount of structural studies on KCNE3, it is not fully understood how KCNE3 TMD behaves structurally and dynamically in more native lipid-bilayer membranes. In this study, we will provide in depth characterization of the conformational properties of KCNE3 TMD in a native-like lipid-bilayer membrane using all-atom molecular dynamics simulations. We will also investigate how TMD mutations that interfere with the KCNQ1 interaction affect KCNE3 conformation in the absence of KCNQ1.

## 2. Methods

### 2.1. Molecular Dynamics Modeling of KCNE3 in Lipid Bilayers

Nanoscale molecular dynamics (NAMD) version 2.14 [12] with the CHARMM36 force field was utilized to perform molecular dynamics simulations on a full-length wild-type KCNE3 (PDB ID: 2M9Z) [7] and KCNE3 in the presence of interaction-impairing mutations (F68A, V72A, and I76A) in a lipid bilayer containing a mixture of POPC and POPG lipids (3:1) [13,14]. The input files of the simulation were created using CHARMM-GUI (http://www.charmm-gui.org, accessed on 21 July 2020) following the previously published methods [10,15]. The MD trajectory data analysis was carried out using visual molecular dynamics software (VMD) 1.9.1 [16]. The membrane bilayer consisting of pre-equilibrated lipids having a surface area of ~12,010.5 Å^2^ was constructed by using membrane builder protocol in CHARMM-GUI [15,17]. The protein was embedded into the lipid-bilayer membrane. Bulk water was then added above and below the membrane by solvating the system into a TIP3 water box. The system was further ionized with KCl to make it neutral following the membrane builder protocol [15,17]. The final accumulated system contained the protein, water, phospholipids and ions. Four independent simulation systems were prepared corresponding to wild-type KCNE3, F68A KCNE3, V72A KCNE3 and I76A KCNE3 in POPC/POPG lipid bilayers and two independent NAMD simulations were carried out for each system. Table 1 shows the simulation system sizes, number of water molecules and number of replicas of all four systems (WT KCNE3, F68A KCNE3, V72A KCNE3 and I76A KCNE3) studied. Six equilibration steps were performed with 2 fs timesteps for 50 ps-200 ps for each simulation system with NAMD using the input files obtained from CHARMM-GUI as instructed in the membrane builder protocol [15,17]. The production runs were carried out from each equilibrated system. The 2 fs timestep was chosen to provide rigid bonds between hydrogen and heavier atoms for numerical stability and accuracy in conserving the energy. In order to obtain a stable simulation, collective variable restraints were used in minimization equilibration inputs to slowly release the system. NAMD simulations were performed for ~200 ns starting from this equilibrated system. Langevin dynamics was used for the simulations. Electrostatic interactions were computed using the Particle-Mesh Ewald algorithm with a 12 Å cutoff distance [18]. Van der Waals interactions were computed with a 12 Å cutoff distance and a switching function to reduce the potential energy function smoothly to zero between 10 and 12 Å. Periodic-boundary conditions were used, and a constant temperature of 303 K and pressure of 1 atm were maintained during the simulation. Equations of motion were integrated with a time step of 2 fs, and trajectory data were recorded in 20 ps-100 ps increments [19].

### 2.2. Analysis of the MD Simulation Data

#### 2.2.1. Calculation of MD Simulation Output Parameters

The equilibration time of the simulation system was eliminated by omitting the first 15 ns of each trajectory of the production run. The structures in the MD trajectory data were aligned with respect to the starting structure after 15 ns of corresponding segments for each MD simulation system for additional analysis. In order to understand the stability and conformational dynamic behavior of KCNE3, the backbone root-mean-square deviation (RMSD), root-mean-square fluctuation (RMSF), Z-distances, total protein–lipid interaction energy, TMD helical tilt angle and width of lipid-bilayer membrane were determined from the aligned trajectory data utilizing the scripts accessible in the visual molecular dynamics (VMD) program package [16]. Different segments were structurally aligned for the RMSD and RMSF calculation of each unique region as noted in each graph. The membrane bilayer thickness was calculated using the VMD Membplugin-1.1 as the distance between two density peaks of the mass density profile of phosphate atoms (PO_4_) along the membrane normal [20]. The heatmaps for the correlation between transmembrane helical tilt angle and the TMD Z-distance with respect to the lipid bilayer membrane center of mass were prepared using Matlab (https://www.mathworks.com, accessed on 23 September 2022). Igor Pro graphics program (https://www.wavemetrics.com, accessed on 12 September 2022) was used to produce images. Miami University Redhawk cluster computing facility was used to run all molecular dynamics simulations.

#### 2.2.2. Helical Bending Angle Calculation

The bending angle (*θ*) of the KCNE3 TMD helix was calculated by measuring the angle between a vector pointing from the alpha carbon of the residue site S74 above to T71 (S74-T71) and the vector from the residue site S74 down to L77 (S74-L77) by following the method reported previously as follows [21,22].

(1) $$\theta =\arccos\frac{\vec{R21}.\vec{R23}}{\left|\vec{R21}\right|\left|\vec{R23}\right|}$$

where $\;\vec{R21}\;$ is the vector pointing above the alpha carbon of the anchor site, $\vec{R23}$ is the vector pointing below the anchor site, $\left|\vec{R21}\right|$ is the magnitude of the vector $\vec{R21}$, and $\left|\vec{R23}\right|$ is the magnitude of the vector $\vec{R23}$.

The bending angle calculation was performed using a PLUMED version 2.7.6 (https://www.plumed.org, accessed on 23 September 2022) on the Miami Redhawk Cluster [22,23,24].

#### 2.2.3. Principal Component Analysis

The conformational flexibility of an α-helix can be resolved into physical deformation components. This can be characterized by bending in two orthogonal planes and twisting along the principal axis [25].The principal component analysis (PCA) was calculated using ProDy [26], and visualized using the NMWiz 1.0 plugin of VMD [16]. The dynamic cross-correlation matrix (DCCM) was determined from the principal component analysis for the top two principal components. DCCM can be used to display time-related information on the relative movement between C_α_ atoms of residue pairs [27]. A positive cross-correlation indicates that the motion of atoms is interrelated in the same direction, while the negative cross-correlation indicates that the residues are inversely correlated [27]. This method was applied to reduce the number of dimensions used to describe the protein motion. The decomposition was obtained by aligning the TMD helix carbon alphas of the ensemble conformation throughout the simulation to the equilibrated protein at the beginning of the simulation. The covariance matrix was calculated based on the standard deviation of the atomic displacements. The vector arrows were drawn on the structure of the KCNE3 using the Normal Mode Wizard function of ProDy and Visualized by VMD version 1.9.1 [16].

## 3. Results

**Figure 1:**
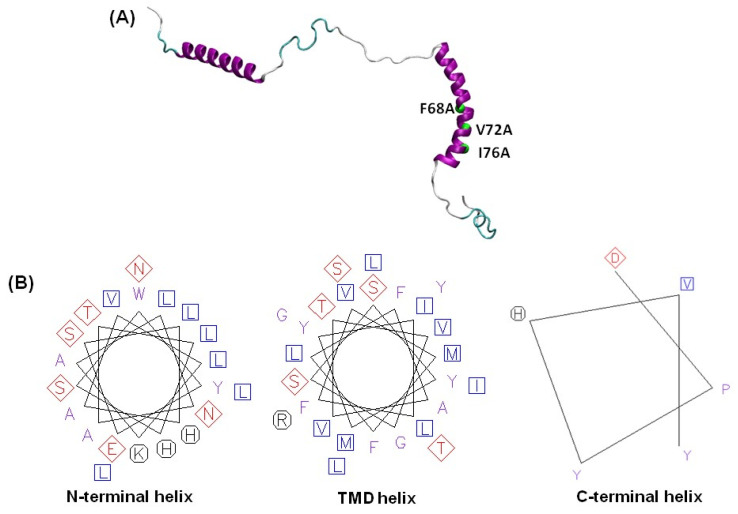
(**A**) A cartoon depiction of KCNE3 NMR structure (PDB ID: 2NDJ) [7]. The interaction-impairing mutants are highlighted by green spheres at alpha carbon positions. (**B**) The helical wheel diagram of helices of KCNE3. The hydrophobic, hydrophilic and positive residues are represented by squares, diamonds and octagons, respectively.

All atom molecular dynamics (MD) simulations are very useful computational methods for studying conformational dynamics of membrane proteins/peptides at the atomic level [19,28,29,30,31]. Here, we employ all atom MD simulations over a period of 200 ns to investigate conformational dynamic properties of the transmembrane domain of KCNE3 in a lipid bilayer membrane consisting of POPC/POPG (3:1). The POPC and POPG lipids are fully miscible. They have phase transition temperatures to the L_α_ liquid crystalline phase below 0 °C and are similar with the most common phospholipids found in mammalian cell membranes [32,33]. The POPC/POPG mixtures are commonly utilized lipid-bilayer-membrane systems to imitate biological membranes for studying membrane proteins using biophysical approaches [7,28]. In a recent publication, we investigated the stability and structural dynamics of wild-type KCNE3 in various lipid-bilayer systems employing all atom molecular dynamics simulations [10]. The study suggested that the transmembrane domain of KCNE3 is more stable and exhibits lower flexibility in comparison to the KCNE3 N- and C-termini in three different membrane bilayer systems: POPC/POPG, POPC alone and DMPC alone. In addition, the results revealed that the transmembrane domain of KCNE3 is embedded within the full membrane width, characterized by amino acid residues S57 and S82 located near the surfaces of lipid-bilayer membrane and water interface while residue A69 is centered within the width of the lipid bilayer. In this study, we characterized the conformational properties of transmembrane domain of KCNE3 in a lipid-bilayer membrane containing a mixture of POPC and POPG lipids in a 3:1 ratio. We further investigated the effect of the KCNQ1 interaction-impairing mutations of KCNE3, including F68A, V72A, I76A, on conformational dynamic properties of TMD and outer regions of KCNE3 in a lipid-bilayer membrane. Figure 1A displays the solution NMR structure of KCNE3 with interaction-impairing mutants highlighted with a green sphere at the alpha carbon, while the helical wheel diagram of helices of KCNE3 are indicated in Figure 1B [7]. The helical wheel diagram indicates that a mix of hydrophobic residues, hydrophilic residues and positively charged residues are located in the N-terminal helix (residues 10-30) and the C-terminal helix (residues 90-95). Conversely, the TMD helix (57-82) is predominantly composed of hydrophobic residues with fewer hydrophilic residues and charged residues.

**Figure 2:**
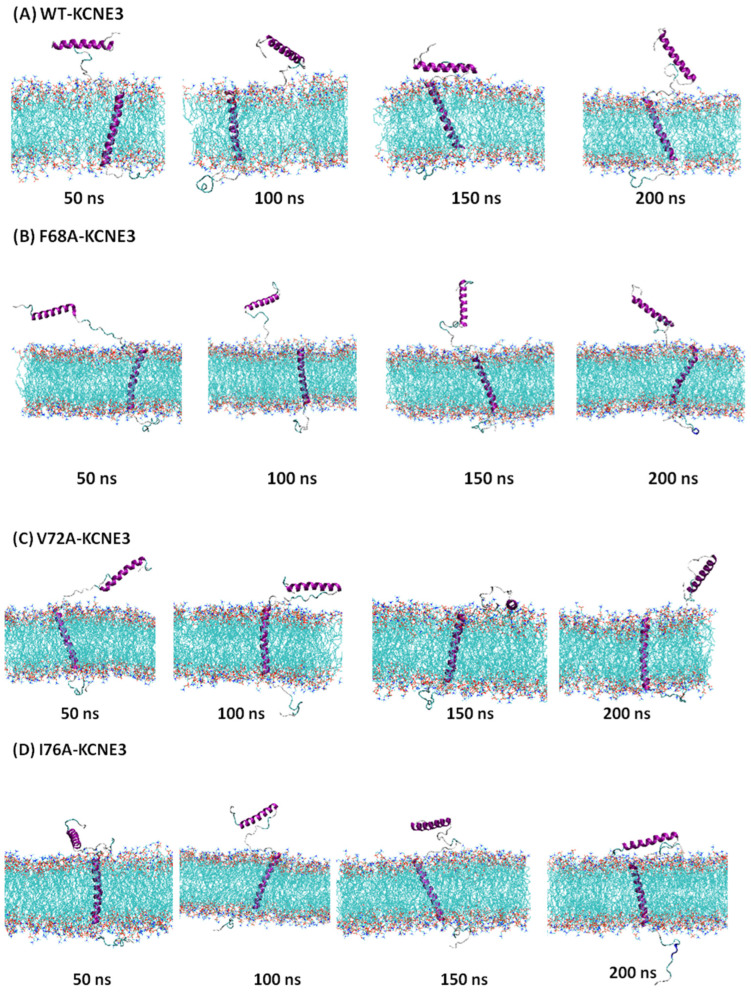
Snapshots of the illustrative MD simulation trajectory data of KCNE3 at 50 ns, 100 ns, 150 ns and 200 ns for wild type KCNE3 (**A**), F68A KCNE3 (**B**), V72A KCNE3 (**C**) and I76A KCNE3 (**D**). Hydrogen atoms and water molecules are omitted for clarity. Images were rendered with VMD [16].

The MD simulation trajectory data obtained over 200 ns were analyzed to investigate structural and conformational dynamics of KCNE3 in POPC/POPG lipid bilayers. Illustrative MD simulation snapshots for KCNE3 WT and mutants at 50 ns, 100 ns, 150 ns and 200 ns are shown in Figure 2. Visual inspection of these snapshots indicates that the KCNE3 N- and C-termini of all four systems (wild-type KCNE3 and mutants) fluctuate freely in solution while the TMD is anchored within the membrane.

### 3.1. Stability of KCNE3 in POPC/POPG Lipid Bilayers

The root-mean-square deviation (RMSD) values are used to compare the average amino acid residue positions in the simulation frame structures to that of the starting structure [34]. The RMSD values are useful in identifying flexible and stable regions of the protein. The average amino acid residue positions of the simulation structure for each frame are compared to the initial structure after aligning based on the position of each unique region. To understand the effect of mutations on the conformational dynamics and stability of the KCNE3 in lipid bilayers, we compare the backbone root-mean-square deviations (RMSD) of different regions of the protein such as transmembrane domain, N-terminus, N-terminal helix, C-terminus and C-terminal helix for each system as shown in Figure S1. The RMSD profile of all KCNE3 proteins suggests that the systems are stable (Figure S1). Comparison of RMSD values from replicates are shown in Figure S2 and Table S1, highlighting the agreement between trajectories for each system. The RMSD data for the different segments of the protein in WT KCNE3 and KCNE3 containing mutations (F68A, V72A, and I76A) suggest that the N-terminal and the C-terminal of KCNE3 have the highest mobility, as characterized by the larger RMSD values. Though, the magnitude of the fluctuations for the C-terminus differs for KCNE3 WT and mutants, with the highest mobility observed for KCNE3 V72A C-terminus (Figures S1 and S2). In contrast, structural elements such as the N-terminal helix, transmembrane domain helix, and C-terminal helix have relatively low fluctuations in all systems. Overall, the RMSD results indicate that the equilibrium dynamics of KCNE3 are not significantly affected due to interaction impairing mutations. 

To understand how the flexibility of specific regions of KCNE3 affects the fluctuations that perturb the protein stability, we quantitatively determined the residue-wise fluctuation in various regions of KCNE3 reconstituted into lipid bilayers. The root-mean square-fluctuation (RMSF) of KCNE3 residues for wild-type KCNE3, KCNE3 F68A, KCNE3 V72A and KCNE3 I76A incorporated into POPC/POPG lipid bilayers are plotted in Figure S3. The RMSF can reveal which areas of the protein structure are the most mobile [34]. The RMSF profile for KCNE3 amino acid residues is similar for the wild-type, F68A, V72A and I76A systems. The TMD, N- and C-terminal helices residues have lower RMSF values when compared to the residues of other segments for all systems. However, the slight variations in the RMSF values are obtained for N- and C-termini residues. Our RMSF data suggest that the presence of the TMD mutations has no significant effect on the flexibility of KCNE3 TMD residues. The comparison of RMSF data obtained from both simulations shows similar behavior of the RMSF curves in both simulations (Figure S4). The RMSF results are consistent with the RMSD results. The RMSD and RMSF measurements suggested that the MD simulations of KCNE3 incorporated into lipid bilayers are stable for all four systems and suitable for further analysis for studying conformational dynamics of KCNE3 TMD.

### 3.2. Conformational Properties of Transmembrane Domain of KCNE3 in POPC/POPG Lipid Bilayers

A recent cryo-EM study of the KCNE3/KCNQ1 complex in nanodiscs captured a significantly different structure in comparison to the previous NMR structure of KCNE3 alone in isotropic bicelles. The RMSD between the two structures is 7.6 Å and indicates structural differences in transmembrane helix curvature [8]. KCNE3 TMD interacts with KCNQ1, and TMD residues F68, V72 and I76 are involved in the interactions. Therefore, we wanted to investigate these residues on KCNE3 alone to determine how alanine substituted mutants (F68A, V72A and I76A) affect the structural dynamics of KCNE3. 

**Figure 3:**
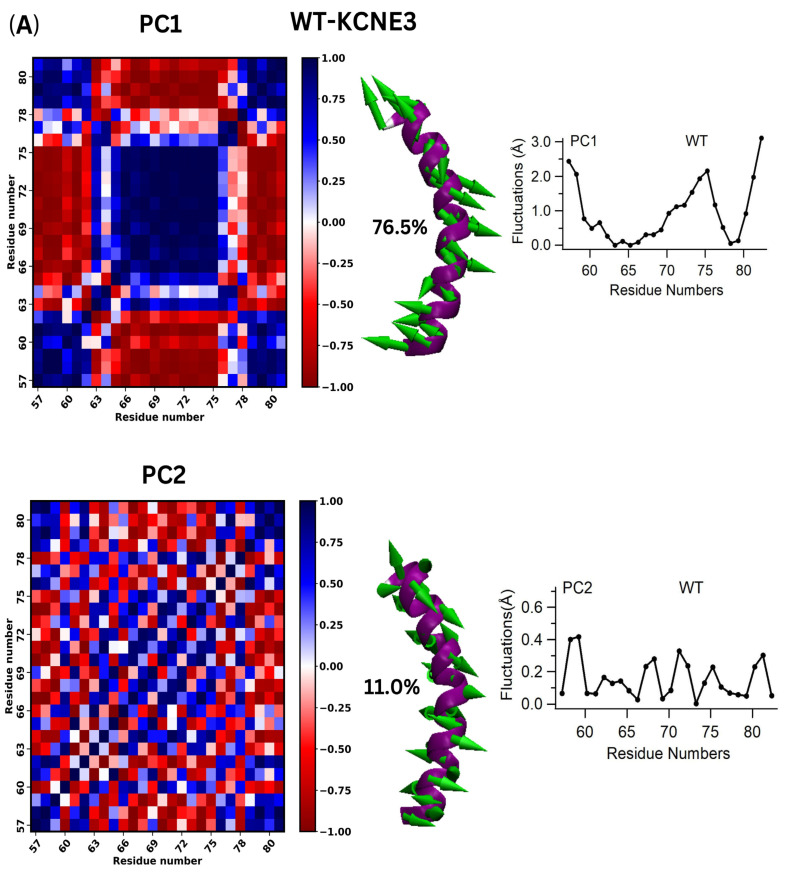

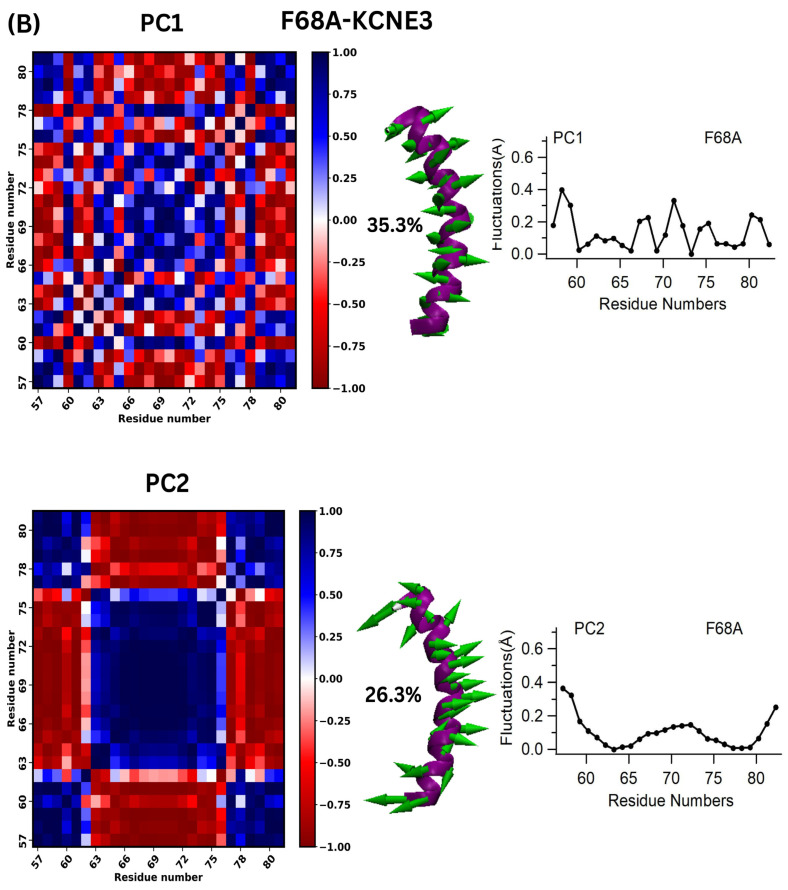

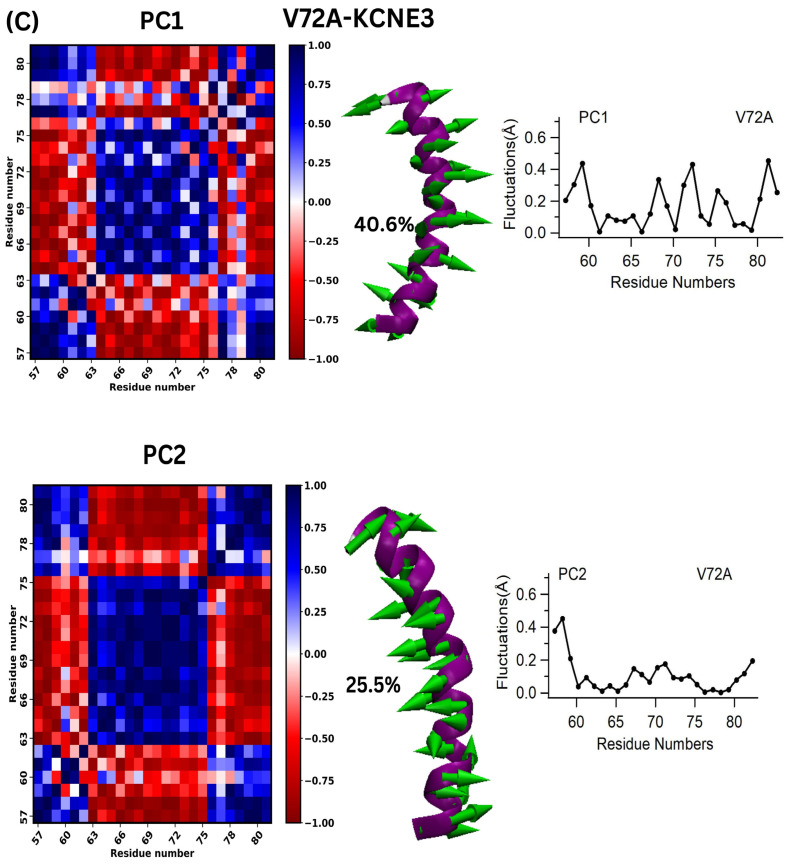

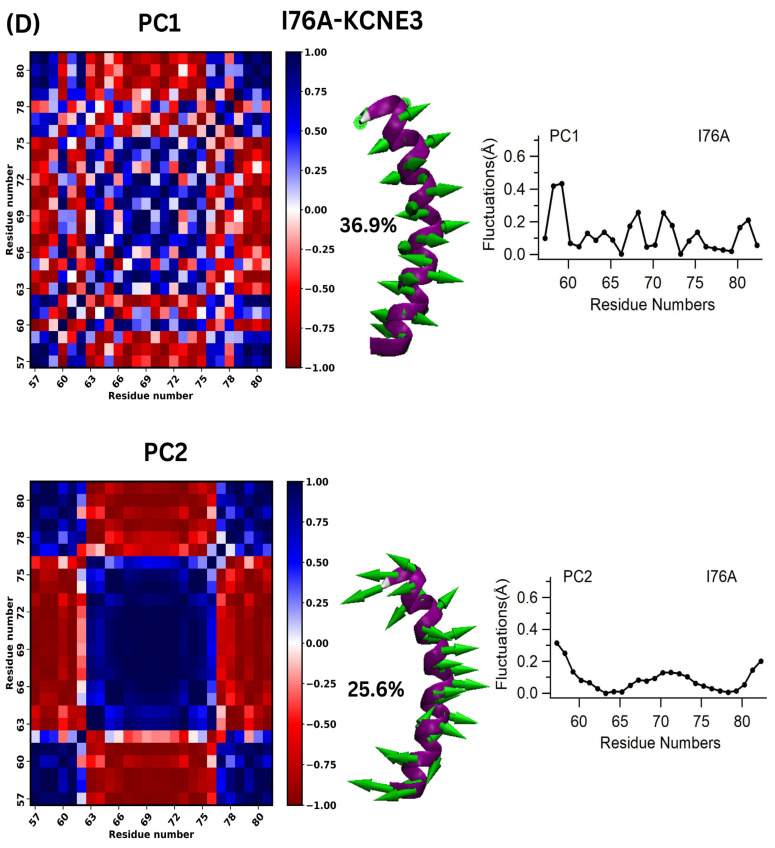
Principal Component Analysis of WT-KCNE3 and mutants. Dynamic cross-correlation matrix (DCCM) computed from PCA analysis for first (PC1) and second (PC2) principal components (left panel). The blue color represents positive correlation, and the red color represents negative correlation on the vertical color bar (left panel). Movies of the fluctuations can be found as Supplementary Materials Movie M1. Vector arrows depicting the motion are mapped onto the protein structure, and corresponding percentage contribution of the first and second principal components are indicated (middle panel). The green arrows represent the direction of the movement of the amino acid residue, and the length represents the relative magnitude of the movement for each residue (middle panel). Individual residue fluctuations (b-factors) are shown for each principal component (right panel). Analysis was carried out for WT KCNE3 TMD (**A**), F68A KCNE3 (**B**), V72A KCNE3 (**C**) and I76A KCNE3 (**D**) incorporated into POPC/POPG lipid bilayers [Supplementary Materials Movie M1].

To understand the conformational motions of the transmembrane domain of KCNE3 in a lipid-bilayer membrane, we performed principal component analysis (PCA). In the calculation, we aligned only the transmembrane domain segment from all atom molecular dynamics simulations (Methods) and applied PCA, since this is the region containing the mutations. The results for WT KCNE3 are plotted in Figure 3A. The top two principal components contributed more than 87% to the overall motions of the system. The dynamic cross-correlation matrix (DCCM) was computed for the top two principal components (the first principal component PC1 and the second principal component vector PC2). The matrices are shown in the left panel of Figure 3A as indicated. When comparing correlation between regions, blue coloring indicates highly correlated motions, red indicates anti-correlated motions, and white indicates no correlation. For WT KCNE3, PC1 corresponds to a stretching of the TMD helix terminal ends, while PC2 corresponds to a rotational movement (Supplementary Materials movies MA1, MA2). Vector arrows depicting the motions for PC1 and PC2 are mapped onto the residues of the KCNE3 TMD structure in Figure 3A (center), and the corresponding percentage contribution for each motion relative to the overall fluctuations is indicated. Together, a strong correlation is noted for three regions of the TMD helix comprising the N-terminal residues (residues 57-62), the C-terminal residues (residues 77-82) and residues in the middle of the helix (residues 63-76). The C- and N- terminal residues move in a correlated fashion relative to each other and anti-correlated relative to the middle region of the TMD helix. The motions in PC2 are more dispersed than in PC1, suggesting more intricate motions. The residue fluctuations (b-factors) are shown for each residue for PC1 and PC2 (Figure 3A, right), as indicated. In PC1, high mobility is observed for C-, N- and middle TMD helix residues. Conversely, an overall lower mobility is observed for these regions in PC2.

Next, we compared the results for wild-type KCNE3 and KCNE3 containing F68A, V72A and I76A mutants to determine how mutations affect the conformational fluctuations of the transmembrane domain region. For the three mutants, PC1 and PC2 accounted for at least 60% of the overall movement, which is reduced compared to WT (Figure 3B–D). Additionally, the dominant modes for the mutants compared to WT are swapped, with the rotational motion characterized as the most dominant mode, PC1 (Supplementary Materials movies MB1-MD1). PC2 for F68A and V72A suggest a bending motion of the helix, while PC2 for I76A shows a different rotational motion (Supplementary Materials movies MB2-MD2). Overall, the correlation between the N-, C- and middle regions of the TMD helix are similar compared to WT. However, the individual residue fluctuations are reduced for the mutants compared to PC1 of WT (Figure 3), suggesting that the mutants may have stabilizing effects. Fluctuations (b-factors) computed from PCA analysis for first principal component (PC1) and second principal component (PC2) for each residue mapped on KCNE3 TMD structure are shown in the Supplementary Materials (Figure S5). Together, these results suggest that the mutations have altered the overall fluctuations and introduced new motions. PCA analysis of the replicate simulations is consistent with these findings (Figure S6).

**Figure 4:**
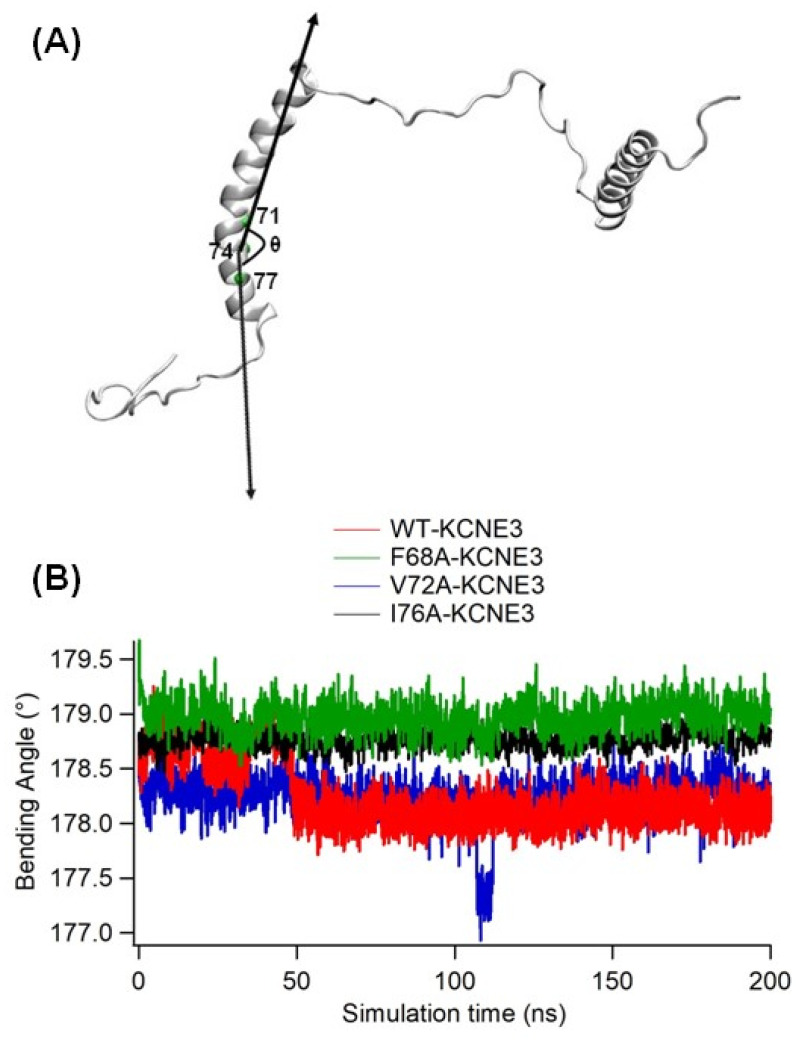
Bending angle calculation of KCNE3 TMD. (**A**) A schematic diagram of the direction of the vectors projected on the KCNE3 TMD used to calculate the bending angle (*θ*) projected on the NMR structure of KCNE3 (PDB ID: 2NDJ). (**B**) The plot of the bending angle of KCNE3 TMD as a function of the simulation time for WT KCNE3, F68A KCNE3, V72A KCNE3 and I76A KCNE3.

Previous solution NMR experiments on KCNE3 in isotropic bicelles suggested that the amino acid residues T71, S74 and G78 of the concave face of the TMD helix were involved in helix bending [7]. In agreement with this finding, PCA analysis suggested that KCNE3 F68A and V72A comprised a small composition of helix bending. To further characterize the conformational properties of the KCNE3 TMD WT and mutants, we directly calculated helix bending angles from the simulations by measuring the angle between the vector pointing from the alpha carbon of S74 to the top of the helix (S74-T71) and the vector pointing from the alpha carbon of S74 to the bottom of the helix (S74-T77) as indicated by Figure 4A. The bending angle was plotted against the time of simulation for wild-type KCNE3 TMD and the KCNE3 TMD containing mutations as shown in Figure 4B. The bending angle for the WT KCNE3 TMD slightly increases from 0 to ~50 ns and remains stable up to 200 ns (Figure 4B). The bending angles for KCNE3 mutants F68A, V72A and I76A are stable throughout the whole simulation (Figure 4B). These results suggest that the wild-type KCNE3 TMD is close to linear in the POPC/POPG bilayer membrane in the timescales simulated. The comparison of the bending angles obtained from both simulations is plotted in the Supporting Information (Figure S7), and the values of bending angles calculated from the independent simulations are similar (Table S2). Together, these results suggest that in the time scales sampled, the TMD helix of KCNE3 WT and mutants does not undergo significant bending.

### 3.3. Effect of KCNE3 Mutations in the Formation of Lipid Bilayers

**Figure 5:**
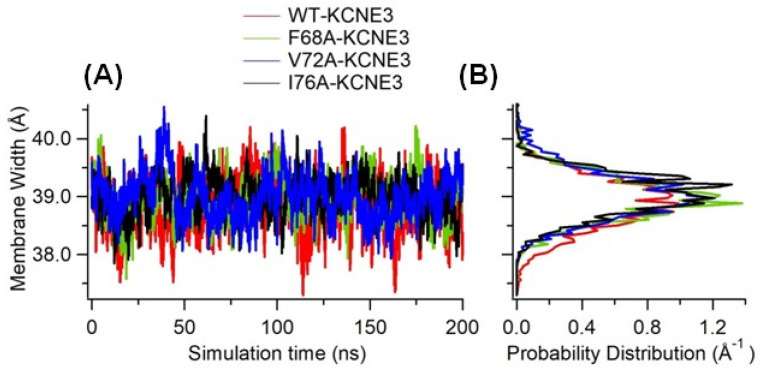
Width of the membrane bilayer embedding KCNE3 protein as a function of simulation time (**A**) and corresponding histogram (**B**) for wild-type KCNE3 (Red), F68A KCNE3 (green), V72A KCNE3 (Blue), and I76A KCNE3 (Black). The x-axis of the histogram plot shows probability distribution. A bin width of 0.047 was used to obtain histogram.

Protein interactions with membrane bilayers can modulate conformational changes of the protein or alter which regions of the protein are embedded in the membrane [35]. Often, the protein/lipid boundary is involved in the protein conformational changes, which can lead perturbations of the adjacent bilayer. The bilayer perturbation for a given conformational change varies as a function of the bilayer thickness [35]. To better understand the effect of the mutations of KCNE3 on the formation of lipid bilayers containing reconstituted KCNE3, we determined the membrane-bilayer width for wild-type KCNE3, F68A KCNE3, V72A KCNE3 and I76A KCNE3 as shown in Figure 5. The membrane width was measured as the distance between two density peaks of the mass density profile of phosphate atoms (PO_4_) along the membrane normal [20]. This is a common method used to determine the membrane width of flat bilayers [36]. The width of the membrane is plotted as a function of simulation time and shown in Figure 5A, while the corresponding probability distribution of the membrane width for each system is shown in Figure 5B. The measured membrane width and distributions are similar for WT KCNE3 and mutants. These data suggest that there is no significant effect of the mutations on the membrane width of the POPC/POPG lipid bilayer in the sampled simulation time. The membrane width measurements are also consistent with the bending angle calculation (Figure 4), suggesting that there is no significant change in the bending angle values due to the presence of mutations.

### 3.4. Topology of KCNE3 with Respect to Lipid Bilayers

**Figure 6:**
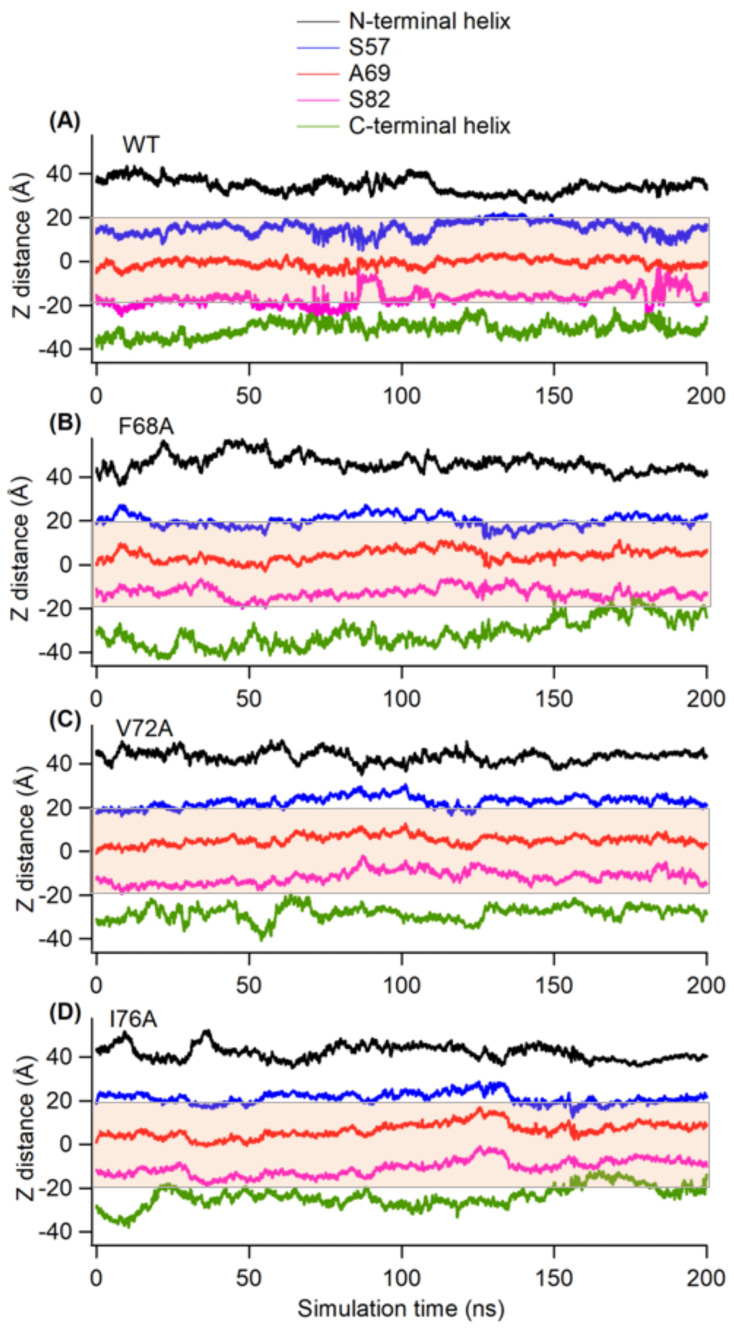
The plot of Z-axis distance (Z-distance) as a function of simulation times for wild-type KCNE3 (**A**), F68A KCNE3 (**B**), V72A KCNE3 (**C**) and I76A KCNE3 (**D**) incorporated into POPC/POPG lipid bilayers. Shaded regions represent the average width of the lipid bilayers.

The conformational changes in the TMD region of KCNE3 may influence the location of different amino acid residues with respect to the lipid-bilayer membrane. Our membrane width data suggested no significant changes in the membrane depth due to the presence of mutations in KCNE3 TMD. Next, we wanted to determine whether there is any influence in the location of the various segments of KCNE3 with respect to the lipid bilayer membrane due to the presence of interaction impairing mutations. The Z-axis distance (Z-distance) of various regions of KCNE3 from the lipid bilayer center of mass was calculated for wild-type KCNE3 and mutants. The orientation of the thickness of the membrane is along the Z-axis with the membrane bilayer center of mass located at Z = 0. The Z-distances of the center of mass of residues corresponding to the bilayer boundaries were computed, including residues S57, A69 and S82, the N-terminal helix and the C-terminal helix, allowing for the determination of the TMD positioning with respect to the bilayer [7]. The plot of Z-distance against the simulation time for the center of mass of different regions is given in Figure 6, allowing for the comparison of WT-KCNE3, F68A, V72A and I76A (Figure 6A–D, respectively). The average Z-distances for the center of mass of different regions’ N-terminal and C-terminal helices and amino residue sites S57, A69 and S82 of the transmembrane domain of KCNE3 are shown in Table S3. The data suggest that the amino acid residue sites S57 and S82 at the terminal ends of TMD reside near the lipid-bilayer surfaces and span the membrane width for wild-type KCNE3 and mutants. The amino acid residue site A69 resides near the lipid-bilayer center for wild-type KCNE3 and mutants, as revealed by the Z-distance around zero. The Z-distance for N- and C-terminal helices varies outside of the range of the membrane width and demonstrates the high degree of flexibility in this region. Together, these results suggest that KCNE3 TMD mutations do not alter the placement/incorporation of the TMD helix in the lipid bilayer. The average Z-distance values calculated from replicate simulations are similar (Table S3).

### 3.5. Probability Density of Helical Tilt Angle and Z-Distance of KCNE3 TMD in Lipid Bilayers

**Figure 7:**
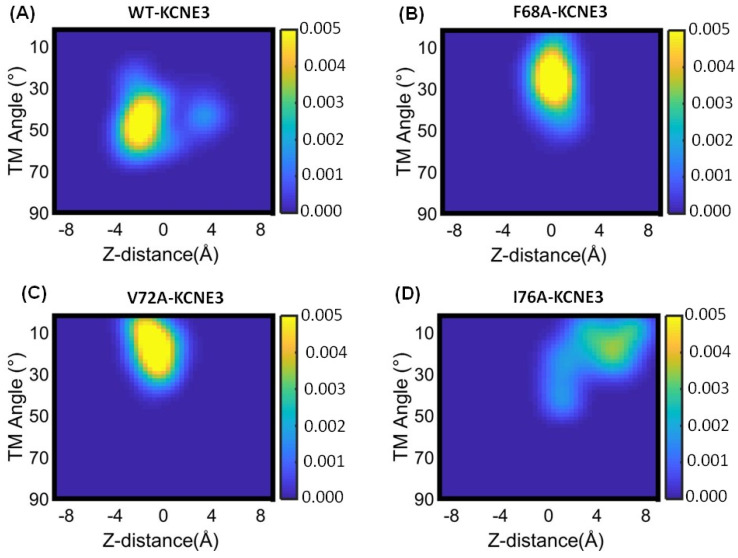
Probability density plot of transmembrane (TM) helical tilt angle against the Z-distance of TMD from the center of mass of lipid bilayer membrane for wild-type KCNE3 (**A**), F68A KCNE3 (**B**), V72A KCNE3 (**C**), and I76A KCNE3 (**D**) in POPC/POPG lipid bilayers. The yellow color indicates the highest probability and blue color represents the lowest probability.

Our results have shown that the membrane-bilayer width is similar for WT KCNE3 and mutants, and the mutations of the TMD do not affect bilayer interactions. We next wanted to understand if mutations cause the TMD helix to reorient within the bilayer and measured the helical tilt angle of the TMD in Figure 7. The correlation of the KCNE3 transmembrane helical tilt with the membrane normal and the Z-distance of the KCNE3 TMD from the lipid bilayer center of mass are plotted for WT-KCNE3 and F68A, V72A and I76A mutants. For the case of wild-type KCNE3, a dominant population exists that is centered on a Z-distance of −2.0 Å and a helical tilt angle of around 35°–60° (Figure 7A). For all three mutants, the helical tilt angle is decreased to values of around 10°–30° (Figure 7B–D). The Z-distance for KCNE3 F68A and V72A is similar around 0 Å (Figure 7B,C). In comparison, the Z-distance is increased for I76A and centered around a value of 6Å. A more dispersed population is observed for I76A KCNE3. These data suggest that the TMD helical tilt occurs for the TMD helix to remain incorporated inside the lipid bilayers. Interestingly, KCNE3 F68A and V72A adopt a more stable configuration, while WT-KCNE3 and I76A do not result in such stabilization. The probability density plots from replicate simulations of the transmembrane helical tilt angle against the TMD Z-distance from the center of mass of lipid-bilayer membrane for wild-type KCNE3 and mutants are shown in Supporting Information Figure S8.

### 3.6. Interaction Energy of Different KCNE3 Regions in Lipid Bilayers

**Figure 8:**
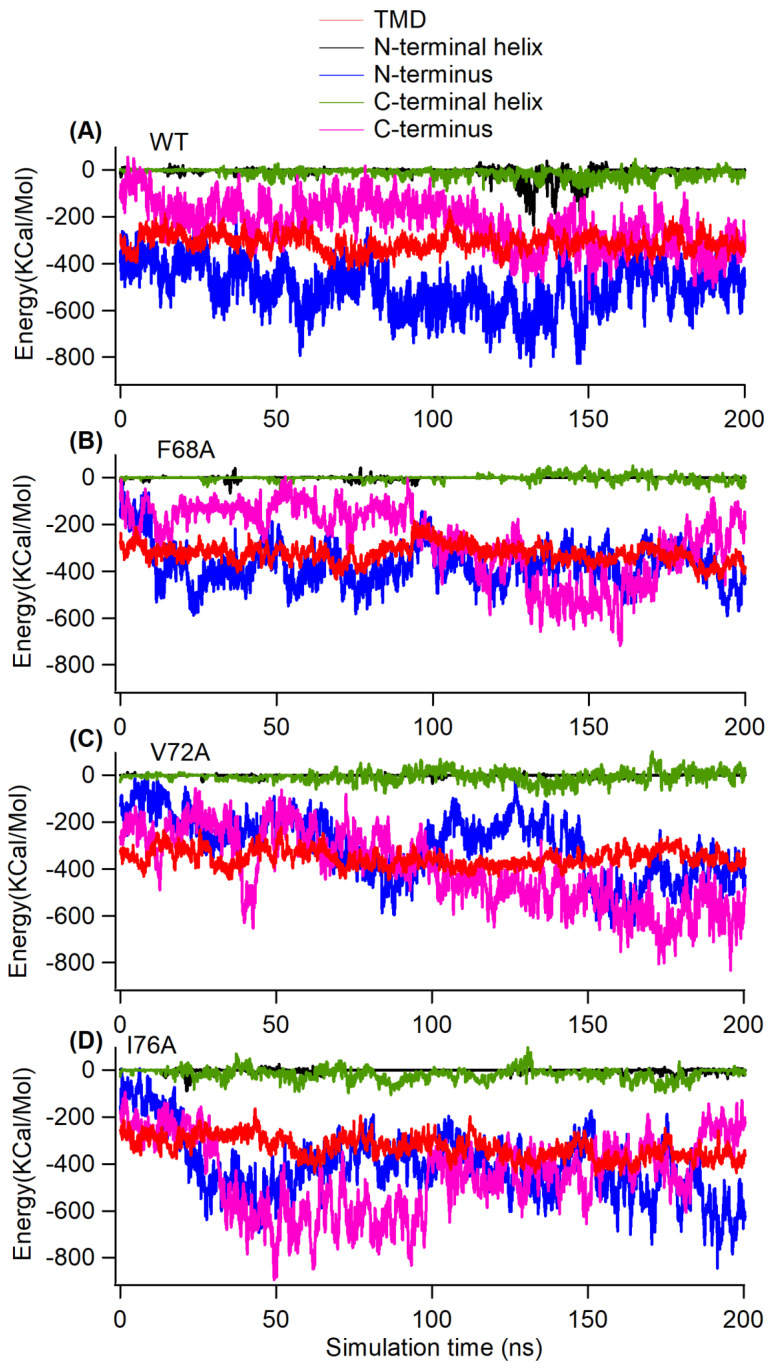
Interaction energy of KCNE3 regions with lipid bilayer membranes as a function of simulation time for KCNE3 WT (**A**), F68A (**B**), V72A (**C**) and I76A (**D**) in POPC/POPG lipid bilayers.

In our analysis, we observed that the mutants were embedded differently in the membrane bilayers (Figure 6). To determine whether TMD mutations affect TMD-lipid bilayer interactions, we calculated the interaction energy (Figure 8). The interaction energy of the TMD with the lipid bilayer is similar for KCNE3 WT and mutants (Figure 8A–D). In all four systems, the interaction energy of the N-terminus, N-terminal helix, and C-terminal helix is very low. Given that these regions reside outside of the membrane bilayers and are highly flexible, they do not strongly interact with the surface of the bilayer. In comparison, the TMD helix, C-terminal helix and C-terminus interact more favorably. The average interaction energy of various regions of KCNE3 calculated from the interaction energy from replicate simulations are compared in Table S4. 

**Figure 9:**
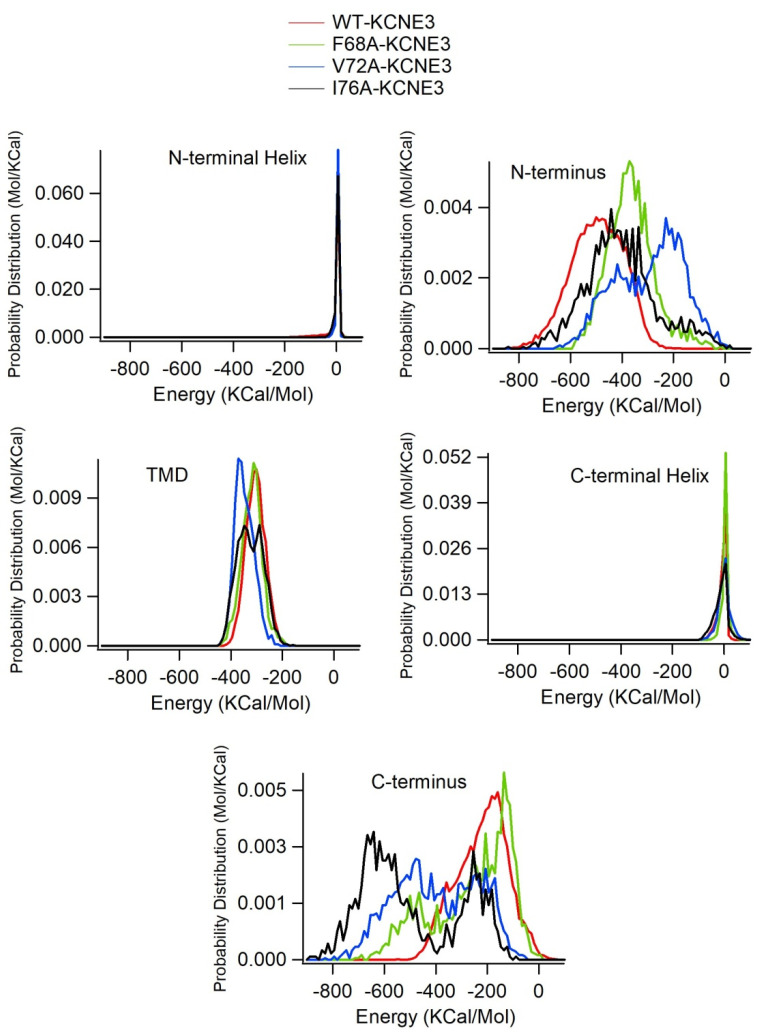
Histogram plots of interaction energy of KCNE3 regions with lipid-bilayer membranes for WT KCNE3, F68A KCNE3, V72A KCNE3 and I76A KCNE3. The histogram plot shows probability distribution. A bin width of 9.4 was used to plot histogram.

To understand the effect of mutations on the interaction energy of KCNE3 in lipid bilayers, we computed the probability distribution from the interaction energy of KCNE3 in lipid bilayers and compared for different mutants (F68A, V72A and I76A) for different segments of KCNE3 as shown in Figure 9. The probability distribution data show that there are no significant variations in the interaction energy of wild-type KCNE3 and different mutants (F68A, V72A and I76A) for TMD, N-terminal and C-terminal helices. However, there are variations among the interaction energies of all four systems for C- and N-termini of KCNE3. The probability distribution data from replicate simulations of interaction energy of all four systems (WT KCNE3, F68A KCNE3, V72A KCNE3 and I76A KCNE3) are shown in Supporting Information (Figure S9). The probability distribution data from both simulations are consistent (Figure 9 and Figure S9). Together, these results suggest that mutations in the TMD do not strongly affect interactions within the lipid bilayer for the TMD, N-terminal and C-terminal helices, but the interactions of N- and C-termini with lipid bilayer are affected due to the presence of mutations.

**Figure 10:**
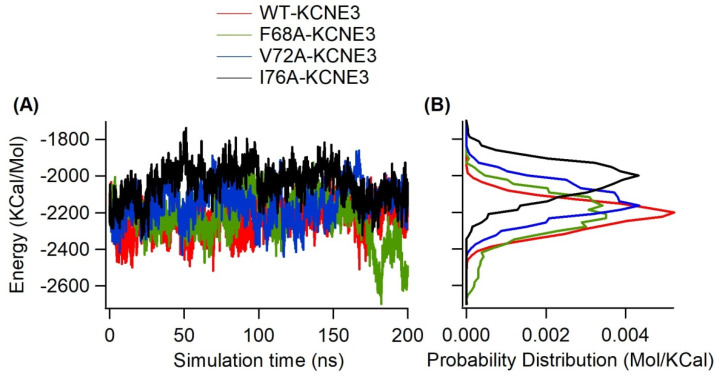
Internal energy of KCNE3 in lipid-bilayer membranes as a function of simulation time for wild-type KCNE3, F68A KCNE3, V72A KCNE3 and I76A KCNE3 (**A**) and corresponding histogram (**B**). The x-axis of the histogram plot shows probability distribution. A bin width of 8.2 was used to generate histogram.

Given the differences in KCNE3 mutants in bilayer interaction, we examined the total internal energy of each KCNE3 system (KCNE3 WT, F68A, V72A, and I76A) as shown in Figure 10. The internal energy represents the minimum total energy of the protein including the electrostatic energy and van der Waals energy contributions associated with the protein reconstituted into lipid bilayers. Figure 10A shows a similar trend for the energy profiles for all four systems. However, the probability distribution of the data (Figure 10B) shows that the total internal energy is shifted to higher energy values for KCNE3 I76A. Together, this suggests that the KCNE3 containing the I76A mutation causes a decrease in the stability of the protein lipid system. This is in agreement with the decreased stability of this mutant system, as observed in the PCA, RMSD and RMSF data (Figure 3, Figures S1 and S2).

## 4. Discussion

Our earlier all-atom MD simulation studies of wild-type KCNE3 over the course of 105 ns revealed that the KCNE3 TMD is more stable and exhibited lower flexibility in comparison to N-terminal and C-terminal of KCNE3 in lipid-bilayer membranes. Additionally, we observed that the TMD spans the width of the membrane bilayer containing the amino acid residue A69 residing near the center of the lipid bilayers and amino acid residues S57 and S82 residing near the surfaces of the lipid-bilayer membrane [10]. In EPR experiments, we observed that the side chain motion of KCNE3 TMD tumbles slowly when compared to that of extracellular termini, and concluded that the amino acid residues of KCNE3 TMD (57-82) are buried inside the lipid bilayers [11]. However, we determined that the extracellular residues are located outside of the lipid-bilayer membrane [11]. A moderately curved α-helical conformation of KCNE3 TMD with residues T71, S74 and G78 residing concave face of curvature was suggested by the solution NMR experimental data of KCNE3 in detergent micelles and isotropic bicelles [7]. Further support of the moderate degree of TMD curvature in lipid bilayers was also provided by double electron–electron resonance (DEER) measurements on the nitroxide spin labeled KCNE3 TMD termini [7]. The authors suggest that the KCNE3 TMD helix curvature is likely stabilized by water transient contact [7]. Since it is well known that the KCNE3 TMD is essential for its function, curvature of the TMD may be a consequence of KCNE3 interactions with the activated-state channel [7]. A previous cryo electron microscopic (Cryo-EM) experiment on the complex of KCNE3 and KCNQ1 in nanodiscs revealed that KCNE3 TMD is pushed against KCNQ1 to stabilize the up conformation of the voltage-sensing domain to lock it in its open configuration [8]. However, the cryo-EM studies of the KCNE3/KCNQ1 complex model containing a truncated version of the KCNE3 (53-95) in nanodiscs. This study revealed that the KCNE3 structure in the KCNE3/KCNQ1 complex deviates from the solution NMR structure of KCNE3 alone in isotropic bicelles, as characterized by a RMSD of 7.6 Å between these two structures [7,8]. This study further suggested an extended form of KCNE3 TMD [8].

Recent functional studies of the KCNE3/KCNQ1 complex suggested that a wider range of amino acid residues, including KCNE3 S57, I61, M65, A69, G73 and I76, that form five helical turns at the center of the transmembrane domain of KCNE3, facilitate the interactions between KCNE3 and the KCNQ1 S1 segment. Additionally, the authors hypothesize that this interaction is needed for the preservation of constitutive activity of the KCNQ1/KCNE3 channel [37]. Despite the significance of KCNE3 TMD, the conformational dynamic properties of KCNE3 are not fully understood. Here, MD simulation data obtained on KCNE3 in POPC/POPG lipid bilayers for 200 ns revealed that the wild-type KCNE3 TMD helix assumes a nearly linear conformation and that the conformation of the TMD is not significantly affected by TMD mutations (F68A, V72A and I76A). The interaction behavior and dynamic properties of the wild-type KCNE3 TMD in POPC/POPG lipid bilayers are more stable and less flexible in comparison to N- and C-termini. These results agree with our previous all-atom MD simulation and CW-EPR results [10,11]. The association of the KCNE3 TMD with lipid bilayers in the presence of mutations is also similar to that of the wild-type KCNE3. This behavior might be due to the fact that the interaction impairing mutations contain hydrophobic amino acid alanine and may favorably interact with the lipid acyl chain [38]. The interaction and dynamics properties of N- and C-termini of KCNE3 with lipid bilayers are variable with stability flexible dynamics in the presence of different interaction-impairing mutations when compared to that of the wild-type KCNE3. In addition, the MD simulation results further revealed that there are no significant changes in the membrane-bilayer widths for the wild-type KCNE3 and KCNE3 containing mutations (F68A, V72A, and I76A). The Z-distance measurements suggested that the wild-type KCNE3 TMD residue site A69 resides near the lipid-bilayer center, and the amino acid residue sites S57 and S82 reside near the membrane-bilayer surfaces. The Z-distance pattern observed for KCNE3 containing mutations is also similar to that of the wild-type KCNE3. The total internal energy of KCNE3 revealed that the wild-type KCNE3 provides more stable simulation and protein-membrane interaction when compared to that of KCNE3 containing mutations.

To examine the reliability of the simulation results, we ran replicate simulations for each system. The results from replicate simulations suggested consistent simulation behavior. Our molecular dynamics simulation data agree with previous computational and experimental biophysical studies of KCNE3 [7,8,10,11,39]. The conformational dynamics behavior of KCNE3 TMD reported in this study is consistent with POPC/POPG lipid-bilayer width and their membrane surface properties [10,11]. However, the structural and conformational dynamic properties of the protein may vary differently depending upon the length and choice of lipids used for simulations [40]. This study complements the biophysical studies of KCNE3 for understanding details of the conformational dynamics of KCNE3 TMD in lipid bilayers.

## 5. Conclusions

All atom molecular dynamics simulations for 200 ns were performed on wild-type KCNE3 reconstituted into a POPC/POPG bilayer membrane environment to investigate the conformational dynamics of KCNE3 TMD. The MD simulation data revealed that the KCNE3 TMD spans the width of the membrane bilayer and does not adopt a significant helical curvature in a POPC/POPG bilayer membrane in the sampled time frame. There is no significant change in the linear helical conformation of KCNE3 TMD due to the presence of interaction-impairing mutations. The TMD of KCNE3 is also more stable with lower flexibility in comparison to the N- and C-termini. The stability and flexibility are similar in the presence of the mutations. The MD simulation data further indicated that the membrane-bilayer widths are similar for wild-type KCNE3 and KCNE3 containing mutations (F68A, V72A, and I76A). Additionally, the MD simulation data revealed that the TMD residue site A69 is close to the lipid-bilayer center and residue sites S57 and S82 reside near the lipid bilayer membrane surfaces for wild-type KCNE3 and KCNE3 containing mutations. These MD simulation data will be complementary to the experimental biophysical results in understanding the details of the conformational dynamic behavior of KCNE3 TMD in bilayer membranes.

## Figures and Tables

**Table 1:** System sizes, number of water molecules and replica of all four systems (WT KCNE3, F68A KCNE3, V72A KCNE3 and I76A KCNE3) studied.

System	System Size	Number of Water	Replica
WT-KCNE3	139.8 Å × 140.2Å × 149.3 Å	56,806	2
F68A-KCNE3	129.3 Å × 129.6 Å × 151.0 Å	48,876	2
V72A-KCNE3	129.5 Å × 129.2 Å × 151.0 Å	48,955	2
I76A-KCNE3	130.9 Å × 129.9 Å × 151.1 Å	48.827	2

## Data Availability

Data are contained within the article and Supplementary Materials.

## Supplementary Materials

The following supporting information can be downloaded at: https://www.mdpi.com/article/10.3390/membranes14020045/s1, Figure S1: Backbone root mean square deviation (RMSD) as a function of simulation time for KCNE3 segments in simulations of WT KCNE3 (A), F68A KCNE3 (B), V72A KCNE3 (C), and I76A KCNE3 (D); Figure S2: Comparison of the backbone root mean square deviations (RMSD) from one simulation (Run 1, black) and a duplicate simulation (Run 2, red) as a function of simulation time; Figure S3: Plot of the root mean square fluctuation (RMSF) of KCNE3 as a function of simulation time for KCNE3 wild type (Red), KCNE3 F68A (green), KCNE3 V72A (Blue), and KCNE3 I76A (Black); Figure S4: Comparison of the backbone root mean square fluctuations (RMSF) from simulation duplicates (Run 1; black and Run 2; red); Figure S5: Fluctuations (b-factors) computed from PCA analysis for first principal component (PC1) (left panel) and second principal component (PC2) (right panel) for each residue mapped on KCNE3 TMD structure are shown for wild type KCNE3, F68A KCNE3, V72A KCNE3, and I76A KCNE3 incorporated into POPC/POPG lipid bilayers. The violet to red color (the bottom color bar) represents the lowest to highest fluctuations (b-factors); Figure S6: Principal Component Analysis (PCA) of KCNE3 WT and mutants for replicate simulations. The dynamic cross-correlation matrix (DCCM) computed from PCA for PC1 and PC2 is shown (left panel). The blue color represents positive correlation and the red color represents negative correlation. Vector arrows are mapped onto the protein structure and the corresponding percentage contribution of the first and second principal components are indicated (center). The green arrows represent the direction of the movement of the amino acid residue and the length represents the magnitude of the movement. The fluctuations (b-factors) of each residue are indicated for each principal component (right panel). The fluctuations of each residue are also mapped on the KCNE3 TMD structure by using color code (bottom panels). The color bar represents the increasing motion violet (lowest) and red (highest). The analysis was performed for WT-KCNE3 (A), F68A-KCNE3 (B), V72A-KCNE3 (C), and I76A-KCNE3 (D); Figure S7: Comparison of the bending angle of KCNE3 TMD from the first simulation (Simulation 1, black) and a replicate simulation (Simulation 2, red) graphed as a function of the simulation time for WT-KCNE3, F68A-KCNE3, V72A-KCNE3, and I76A-KCNE3; Figure S8: Probability density plot from Simulation 2 of transmembrane (TM) helical tilt angle against the Z-distance of TMD from the center of mass of lipid bilayer membrane for wild type KCNE3 (A), F68A KCNE3 (B), V72A KCNE3 (C), and I76A KCNE3 (D) in POPC/POPG lipid bilayers. The yellow color indicates the highest probability and blue color represents the lowest probability; Figure S9: Histogram plots from Simulation 2 of interaction energy of KCNE3 segments with lipid bilayer membranes for WT KCNE3, F68A KCNE3, V72A KCNE3 and I76A KCNE3.; Table S1: Comparison of Average RMSD calculated for different sections of wild type KCNE3 and the KCNE3 in the presence of mutations (F68A, V72A, I76A) from the RMSDs shown in Figures S1 and S2 for Simulation 1 and Simulation 2; Table S2: Comparison of the average bending angles calculated from the bending angles shown in Figure S2; Table S3: Comparison of Average Z-distance calculated from the Z-distance from Simulation 1 and Simulation 2; Table S4: Comparison of Average interaction energy calculated from the interaction energy from Simulation 1 and Simulation 2; Movie M1: Movie of the fluctuations mapped onto the residues of KCNE3 TMD structure for the PC1 and PC2 for WT KCNE3 (MA1, MA2), F68A KCNE3 (MB1, MB2), V72A KCNE3 (MC1, MC2), and I76A KCNE3 (MD1, MD2). These movies are prepared from the Simulation 1 PCA data.

## Abbreviations

MD, molecular dynamics; NAMD, nanoscale molecular dynamics simulation; VMD, visual molecular dynamics; RMSD, root-mean-square deviation; RMSF, root-mean-square fluctuation; PCA, principal component analysis; LMPC, lyso-myristoylphosphatidyl choline; DMPC, 1,2-dimyristoyl-sn-glycero-3-phosphocholine; DHPC, dihexanoylphosphatidylcholine; DMPG, dimyristoylphosphatidylglycerol; POPC, 1-palmitoyl-2-oleoyl-sn-glycero-3-phosphocholine; POPG, 1-palmitoyl-2-oleoyl-sn-glycero-3-phospho-(1′-rac-glycerol) (sodium salt); TMD, transmembrane domain; EPR, electron paramagnetic resonance; NMR, nuclear magnetic resonance; DEER, double electron–electron resonance.
