# Supplementary material for: Studying Conformational Properties of Transmembrane Domain of KCNE3 in a Lipid Bilayer Membrane Using Molecular Dynamics Simulations
Source: Membranes (Basel). 2024 Feb 4;14(2):45. doi: 10.3390/membranes14020045 (PMC10890134; doi:10.3390/membranes14020045)
Supplement: Supplementary file 1 [file membranes-14-00045-s001.zip › KCNE3 Manuscript_Supporting Information 12282023.pdf]

Supporting Information for:

Studying Conformational Properties of Transmembrane Domain of KCNE3 in a Lipid Bilayer Membrane Using Molecular Dynamics Simulations

Anna Clara Miranda Moura[1], Isaac K. Asare[1], Mateo Fernandez Cruz[1], Antonio Javier Franco Aguado[1], Kaeleigh Dyan Tuck[1], Conner C. Campbell[1], Matthew W. Scheyer[1], Ikponwmosa Obaseki[2], Steve Alston[1], Andrea N. Kravats[2], Charles R. Sanders[3], Gary A. Lorigan[2], Indra D. Sahu[1,2*]

[1] Natural Science Division, Campbellsville University, Campbellsville, KY 42718, USA;
[2] Department of Chemistry and Biochemistry, Miami University, Oxford, OH 45056, USA
[3] Department of Biochemistry and Center for Structural Biology, Vanderbilt University, Nashville, TN 37232, USA
* Correspondence: idsahu@campbellsville.edu, Tel.: (270) 789-5597

Contents:

Figure S1
Figure S2
Figure S3
Figure S4
Figure S5
Figure S6
Figure S7
Figure S8
Figure S9
Table S1
Table S2
Table S3
Table S4
Movie M1

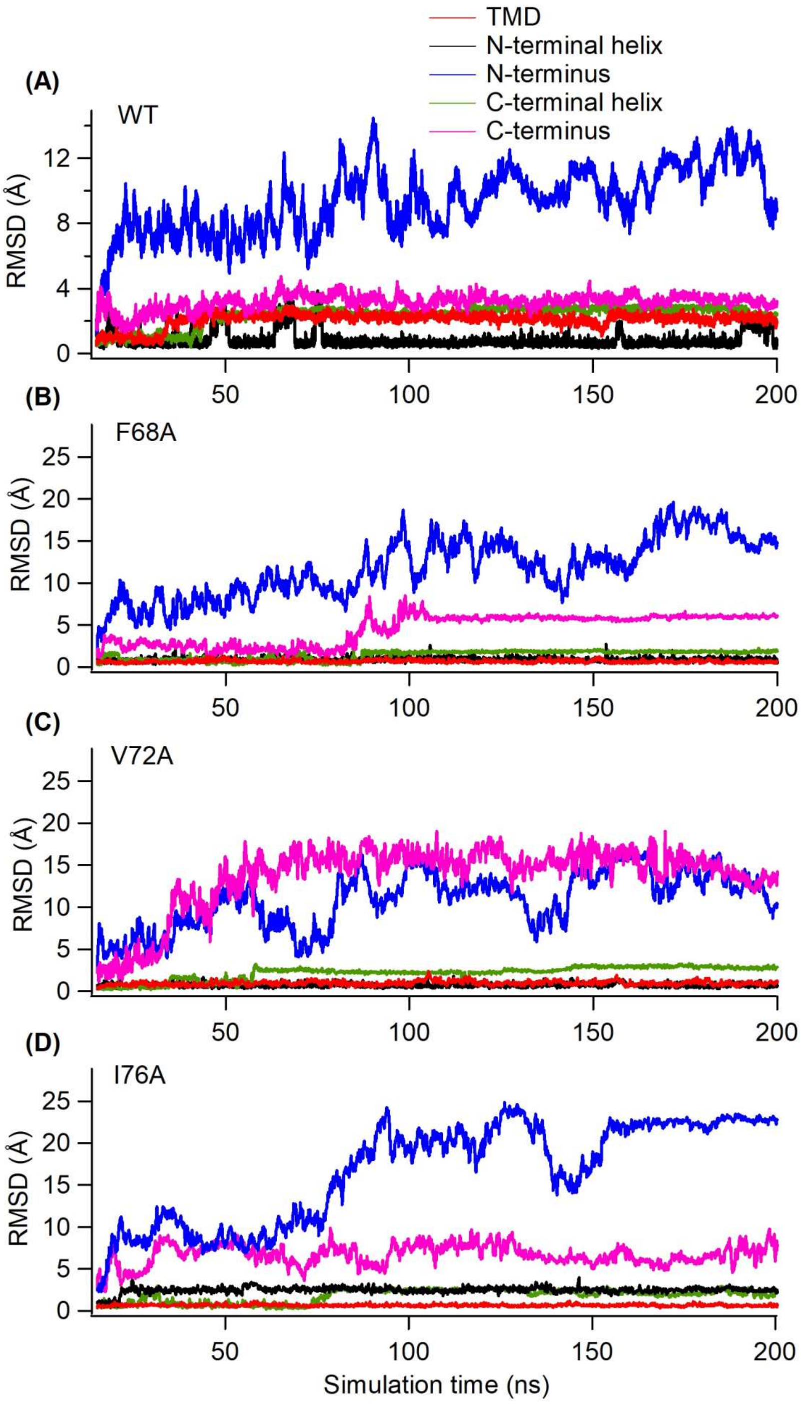

FigureS1. Backbone root mean square deviation (RMSD) as a function of simulation time for KCNE3 segments in simulations of WT KCNE3 **(A)**, F68A KCNE3 **(B)**, V72A KCNE3 **(C)**, and I76A KCNE3 **(D)**. The indicated segments were structurally aligned for the RMSD calculation of each unique region.

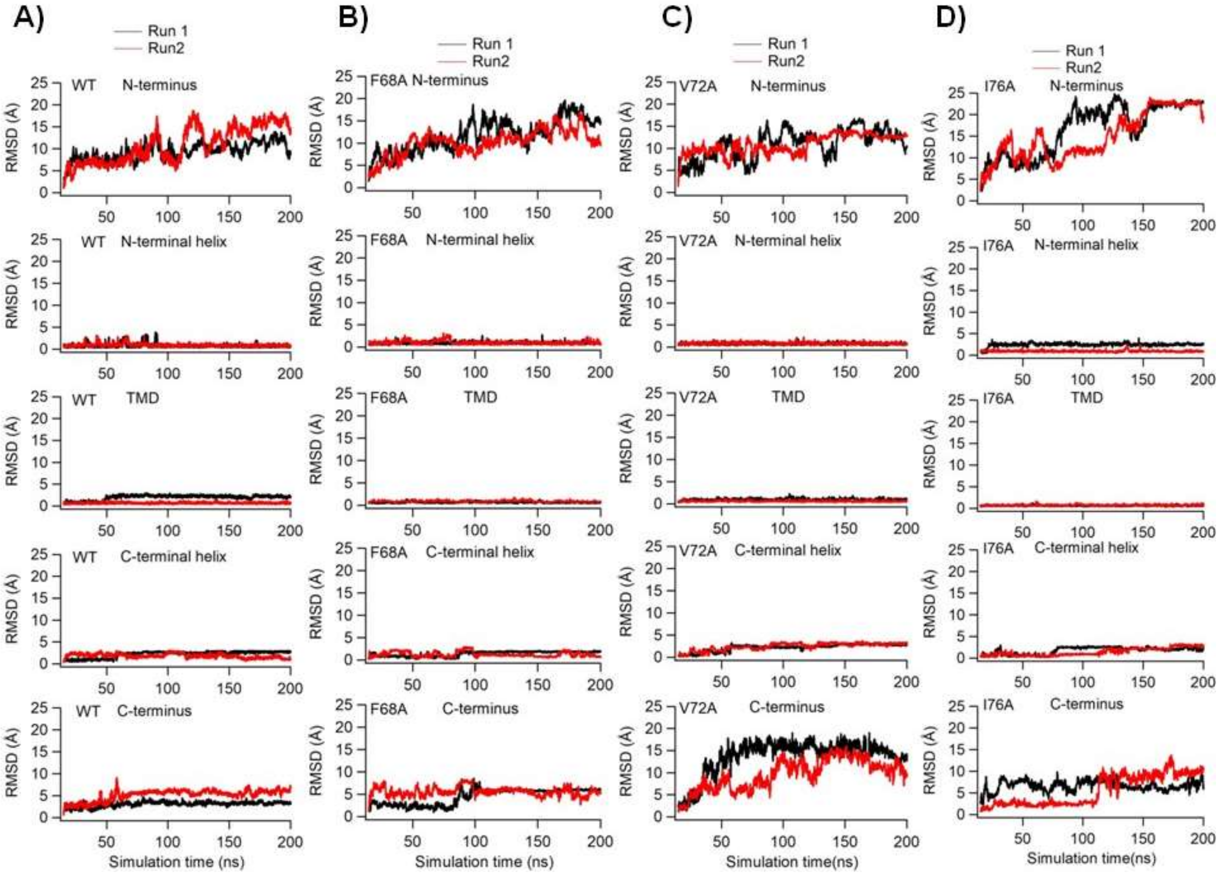

Figure S2: Comparison of the backbone root mean square deviations (RMSD) from one simulation (Run 1, black) and a duplicate simulation (Run 2, red) as a function of simulation time. The RMSD of different KCNE3 segments is measured for WT KCNE3 **(A)**, F68A KCNE3 **(B)**, V72A KCNE3 **(C)**, and I76A KCNE3 **(D)**.

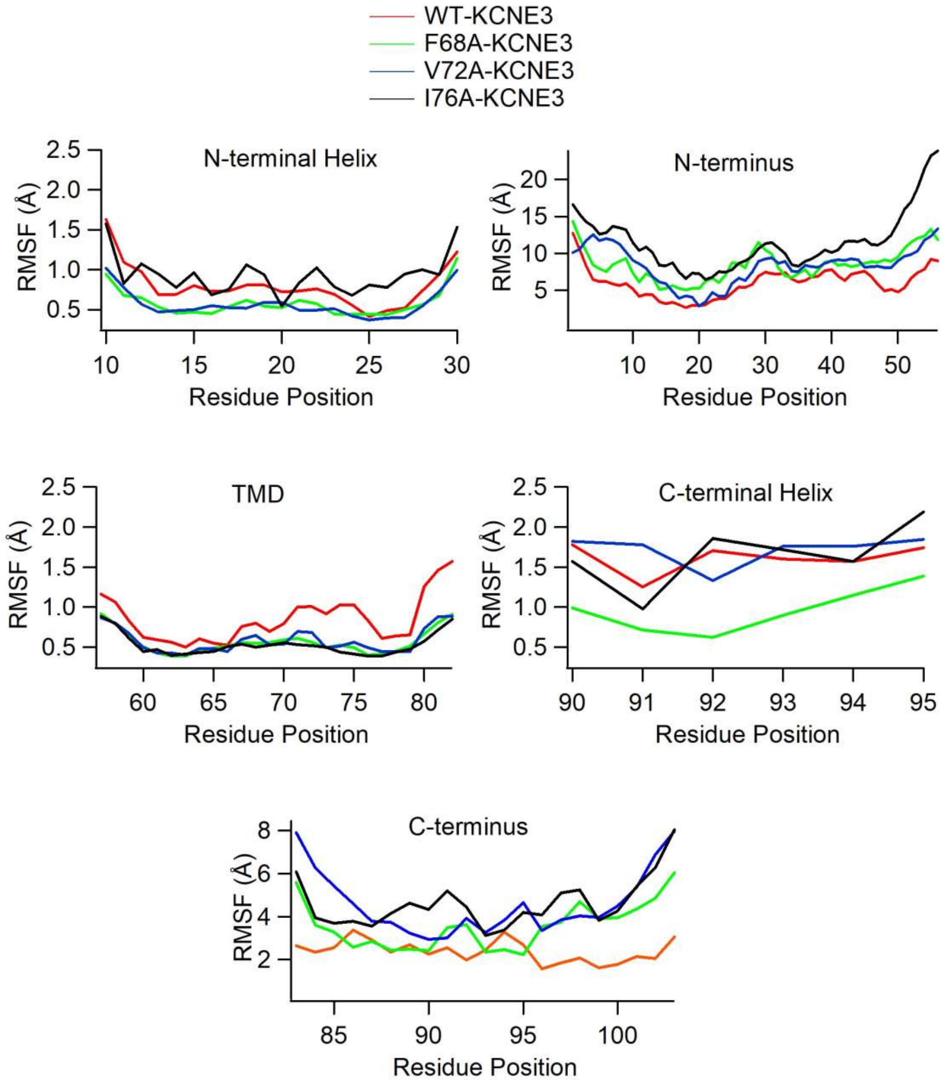

Figure S3. Plot of the root mean square fluctuation (RMSF) of KCNE3 as a function of simulation time for KCNE3 wild type (Red), KCNE3 F68A (green), KCNE3 V72A (Blue), and KCNE3 I76A (Black). The indicated segments were structurally aligned for the RMSF calculation of each unique region. Expanded Y-axis scales were used for N-terminus and C-terminus plots to cover the full range of RMSF values.

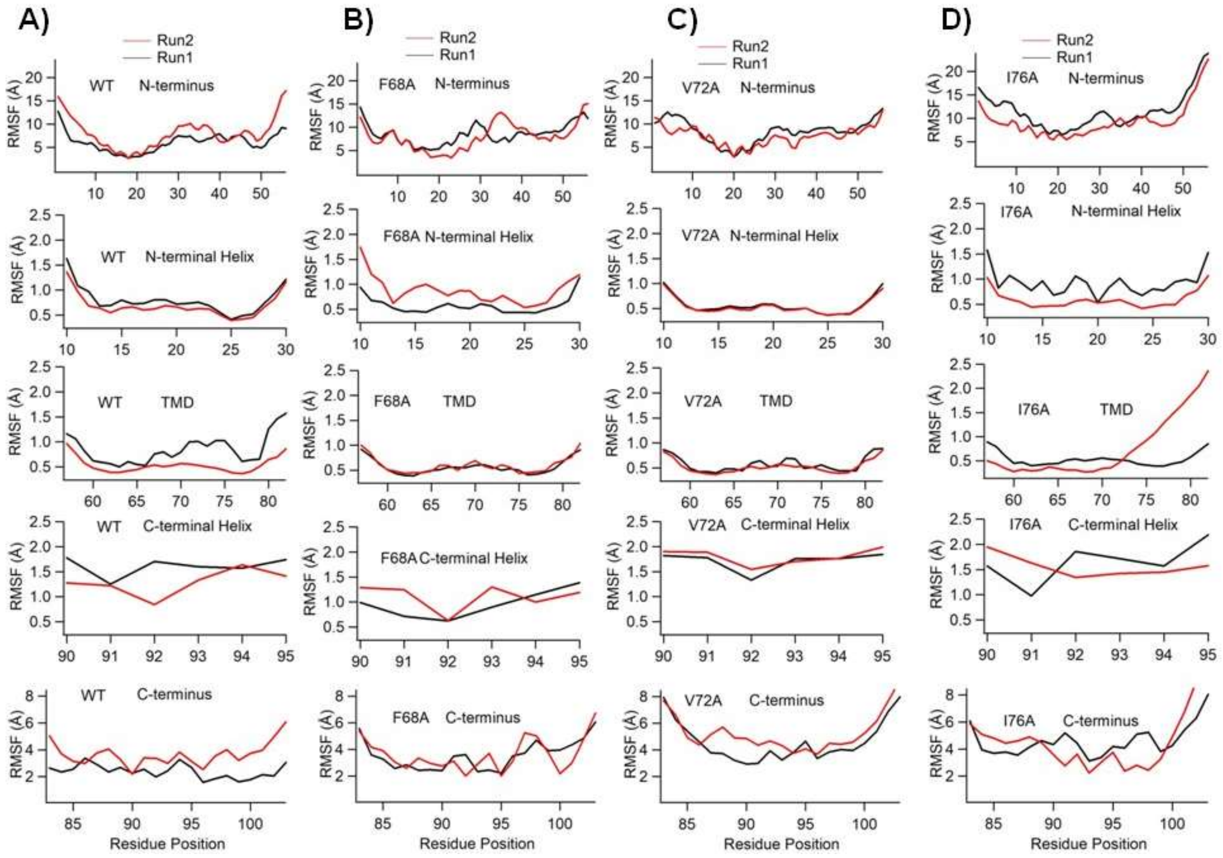

Figure S4. Comparison of the backbone root mean square fluctuations (RMSF) from simulation duplicates (Run 1; black and Run 2; red). RMSF of unique KCNE3 segments was calculated for WT-KCNE3 **(A)**, F68A-KCNE3 **(B)**, V72A-KCNE3 **(C)**, and I76A-KCNE3 **(D)**. Expanded Y-axis scales were used for N-terminus and C-terminus plots to cover the full range of RMSF values.

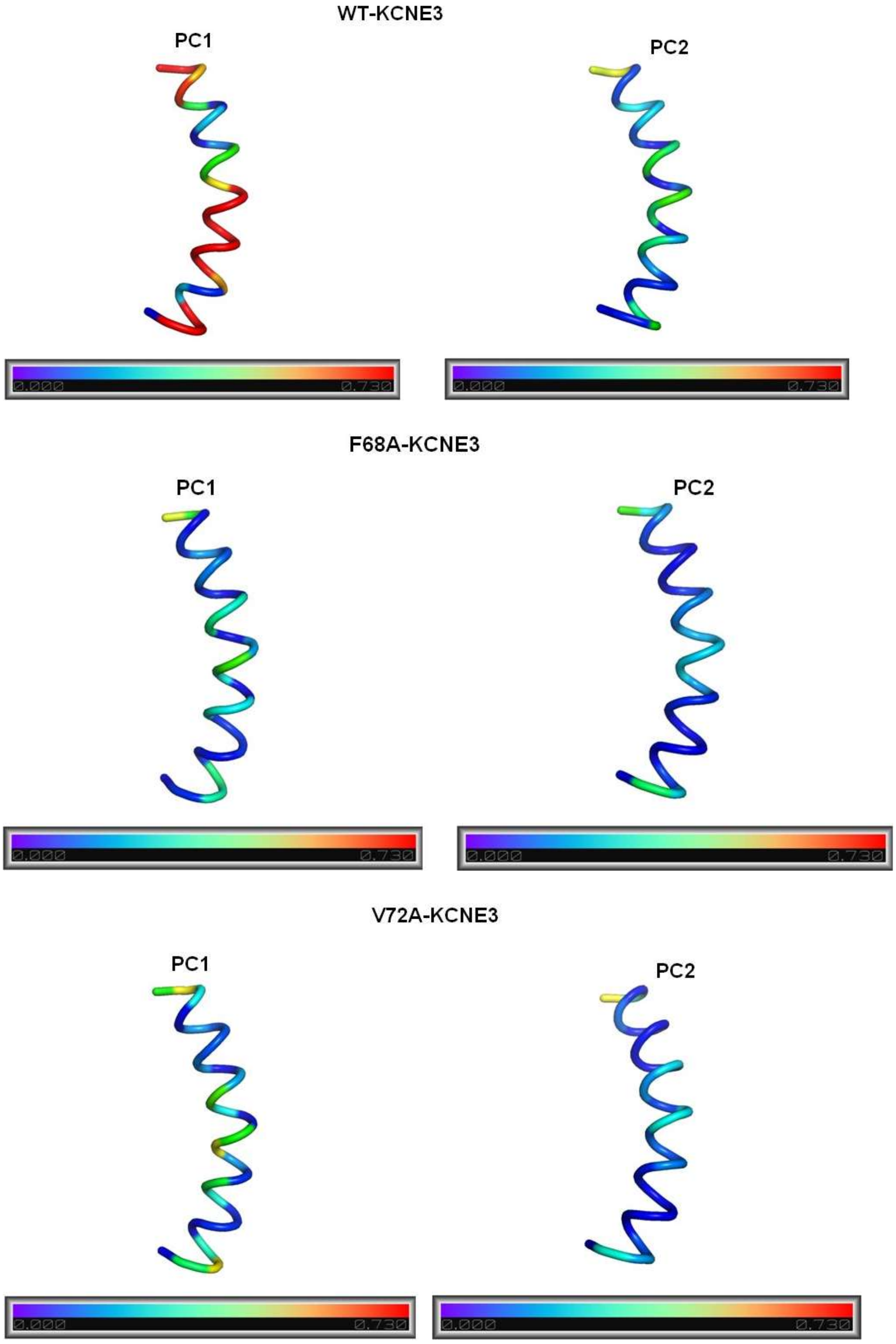

WT-KCNE3
PC1
PC2
0.000
0.730
0.000
0.730
F68A-KCNE3
PC1
PC2
0.000
0.730
0.000
0.730
V72A-KCNE3
PC1
PC2
0.000
0.730
0.000
0.730

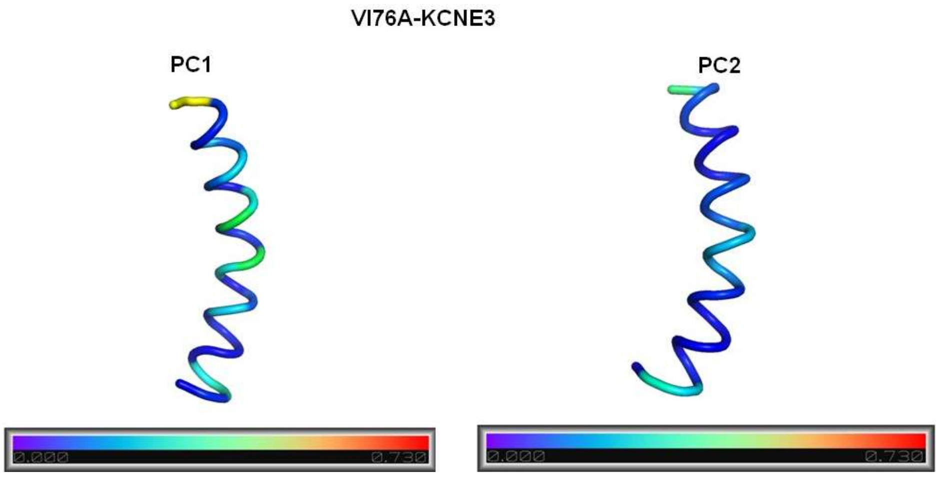

Figure S5. Fluctuations (b-factors) computed from PCA analysis for first principal component (PC1) (left panel) and second principal component (PC2) (right panel) for each residue mapped on KCNE3 TMD structure are shown for wild type KCNE3, F68A KCNE3, V72A KCNE3, and I76A KCNE3 incorporated into POPC/POPG lipid bilayers. The violet to red color (the bottom color bar) represents the lowest to highest fluctuations (b-factors). The image was prepared using PyMOL software (https://pymol.org/2/).

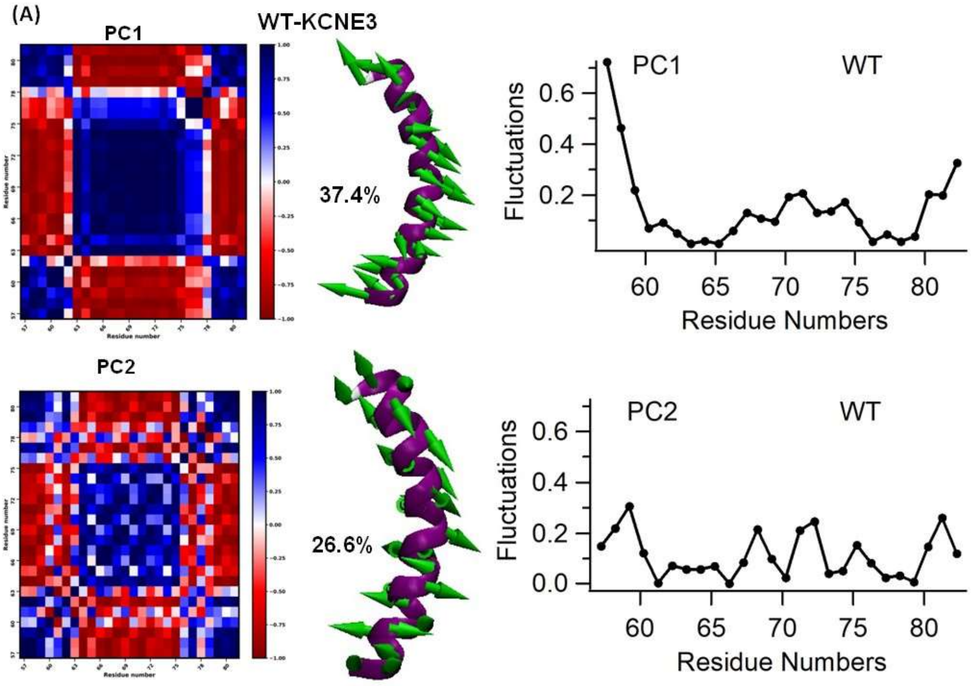
(A)
WT-KCNE3
PC1
37.4%
PC1
WT
Fluctuations
Residue Numbers
PC2
26.6%
PC2
WT
Fluctuations
Residue Numbers

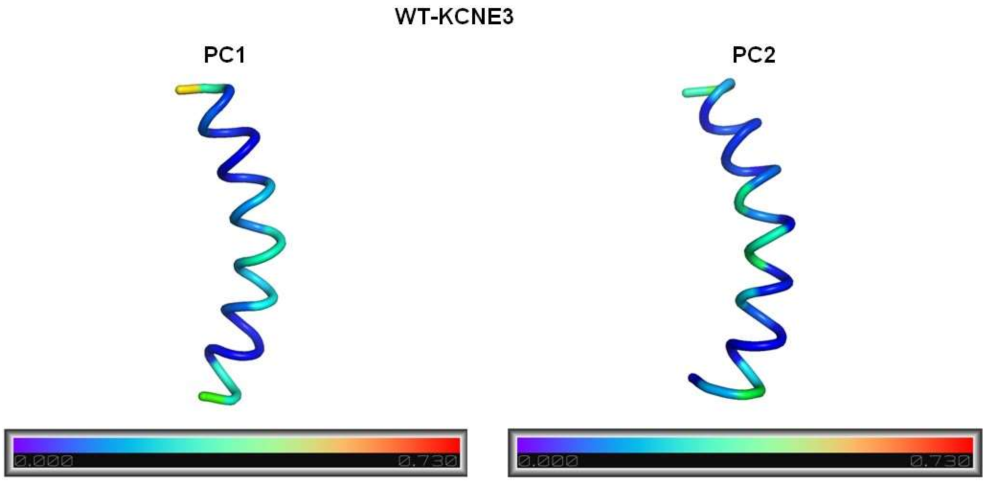
WT-KCNE3
PC1
PC2
0.000
0.730
0.000
0.730

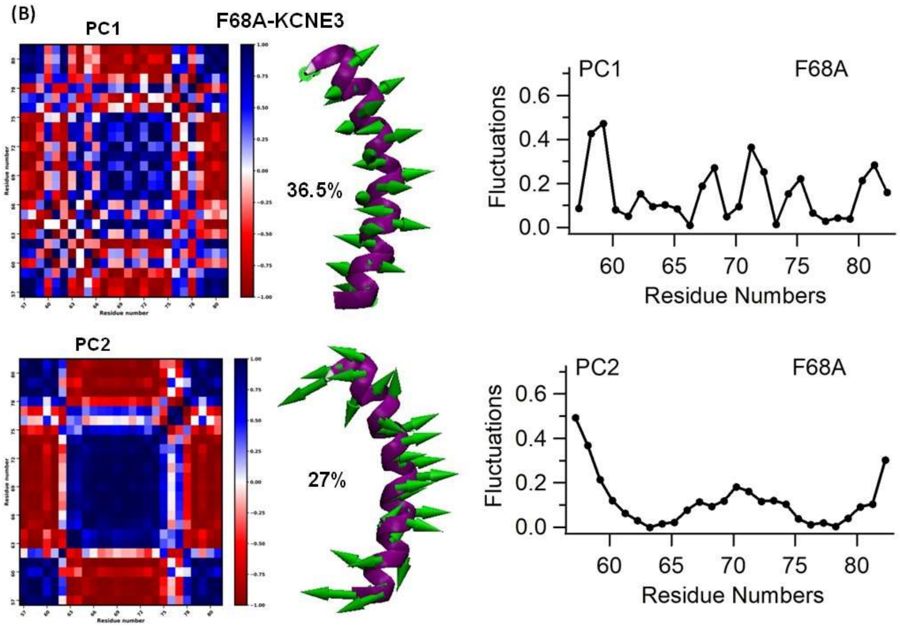
(B)
PC1
F68A-KCNE3
36.5%
PC2
27%
PC1
F68A
Fluctuations
0.0
0.2
0.4
0.6
60
65
70
75
80
Residue Numbers
PC2
F68A
Fluctuations
0.0
0.2
0.4
0.6
60
65
70
75
80
Residue Numbers

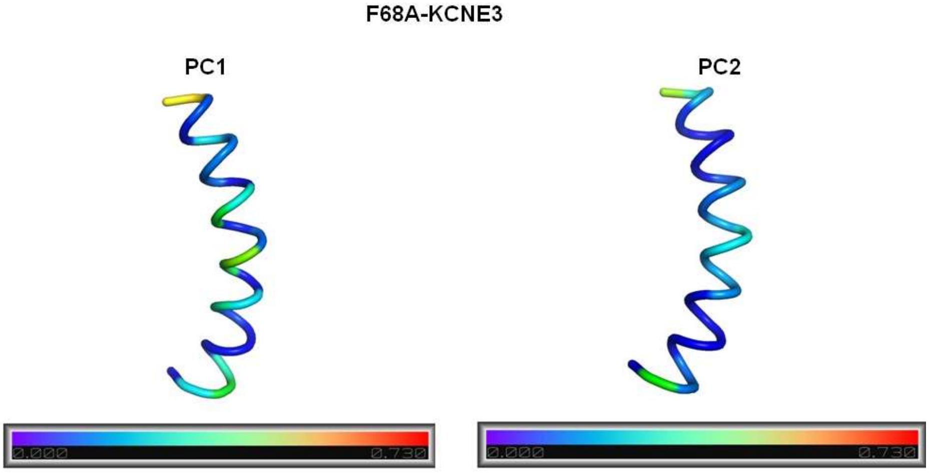
F68A-KCNE3
PC1
PC2
0.000
0.730
0.000
0.730

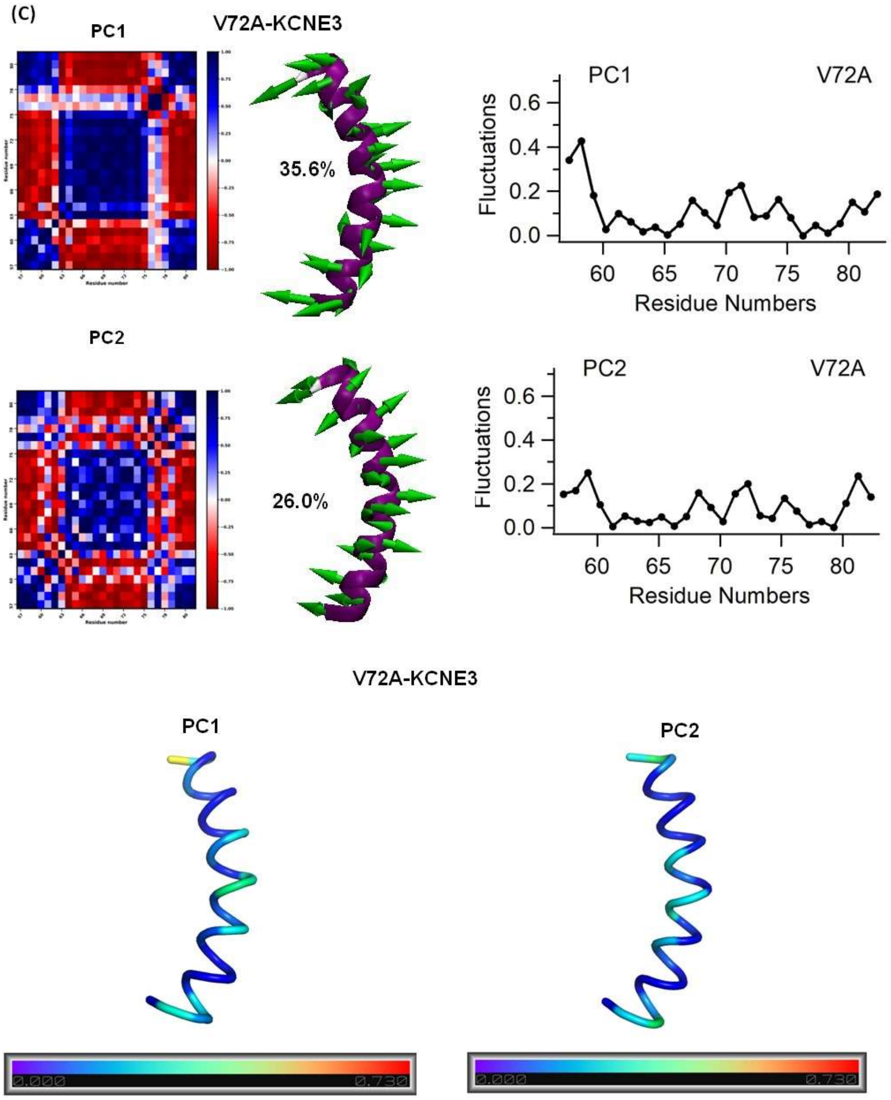
(C)
PC1
V72A-KCNE3
35.6%
PC1
V72A
Fluctuations
0.6
0.4
0.2
0.0
60
65
70
75
80
Residue Numbers
PC2
26.0%
PC2
V72A
Fluctuations
0.6
0.4
0.2
0.0
60
65
70
75
80
Residue Numbers
V72A-KCNE3
PC1
PC2
0.000
0.730
0.000
0.730

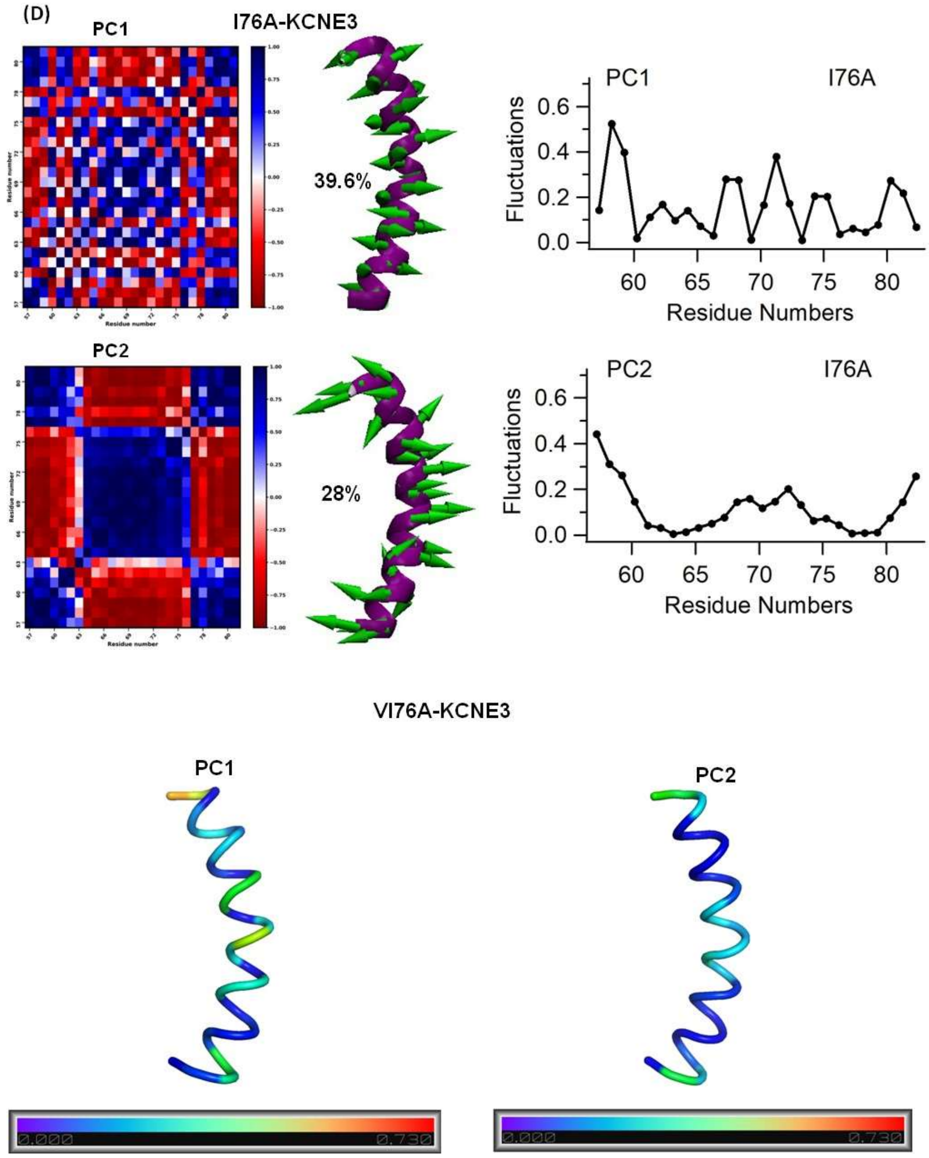

Figure S6. Principal Component Analysis (PCA) of KCNE3 WT and mutants for replicate simulations. The dynamic cross-correlation matrix (DCCM) computed from PCA for PC1 and PC2 is shown (left panel). The blue color represents positive correlation and the red color represents negative correlation. Vector arrows are mapped onto the protein structure and the corresponding percentage contribution of the first and second principal components are indicated (center). The green arrows represent the direction of the movement of the amino acid residue and the length represents the magnitude of the movement. The fluctuations (b-factors) of each residue are indicated for each principal component (right panel). The fluctuations of each residue are also mapped on the KCNE3 TMD structure by using color code (bottom panels). The color bar represents the increasing motion violet (lowest) and red (highest). The analysis was performed for WT-KCNE3 **(A)**, F68A-KCNE3 **(B)** , V72A-KCNE3 **(C),** and I76A-KCNE3 **(D).**

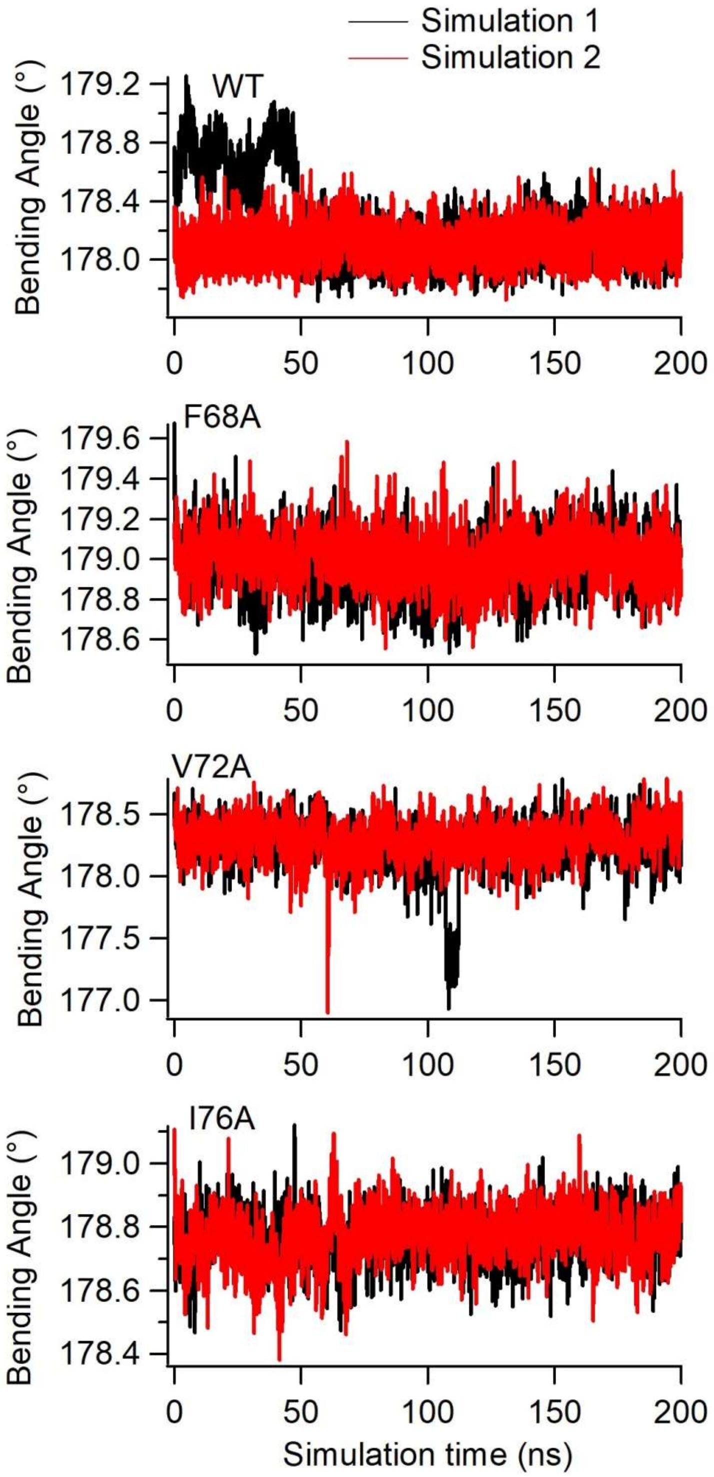

Figure S7. Comparison of the bending angle of KCNE3 TMD from the first simulation (Simulation 1, black) and a replicate simulation (Simulation 2, red) graphed as a function of the simulation time for WT-KCNE3, F68A-KCNE3, V72A-KCNE3, and I76A-KCNE3.

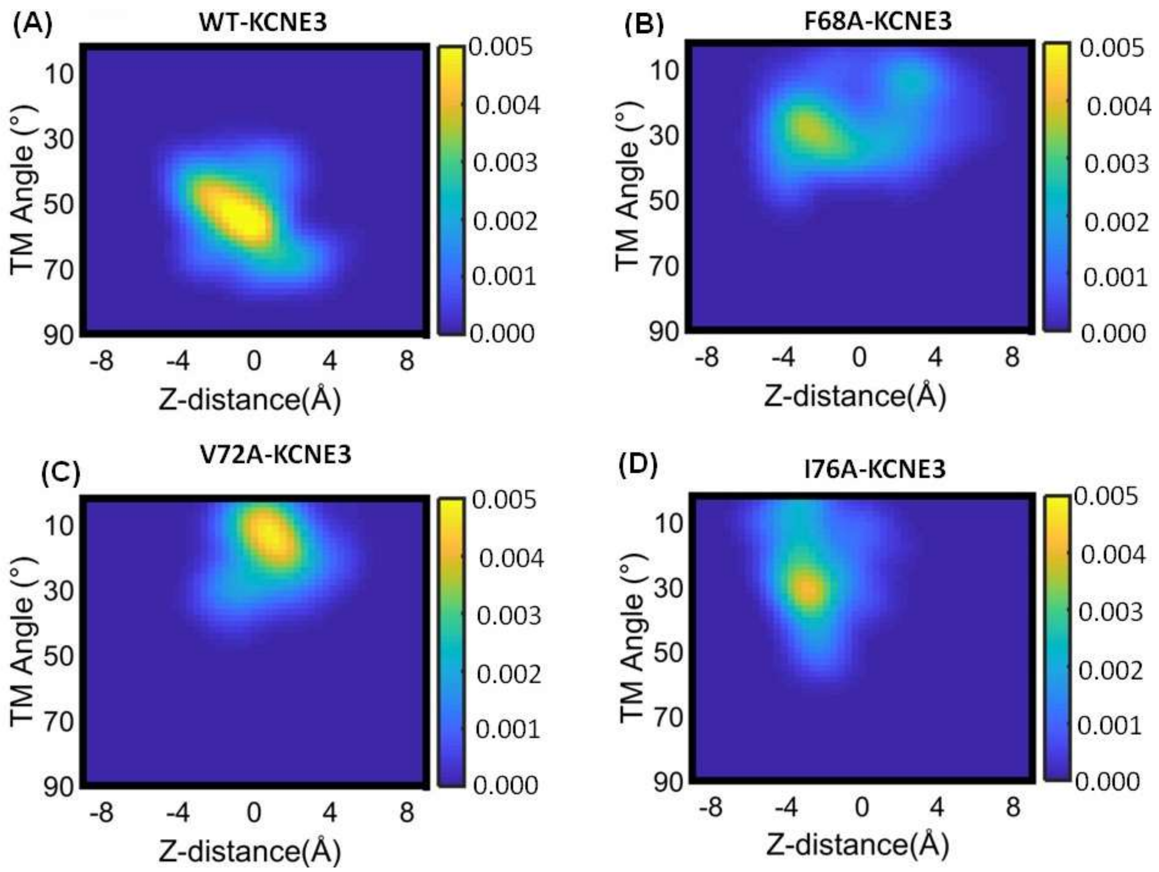

Figure S8. Probability density plot from Simulation 2 of transmembrane (TM) helical tilt angle against the Z-distance of TMD from the center of mass of lipid bilayer membrane for wild type KCNE3 **(A)**, F68A KCNE3 **(B)**, V72A KCNE3 **(C)**, and I76A KCNE3 **(D)** in POPC/POPG lipid bilayers. The yellow color indicates the highest probability and blue color represents the lowest probability.

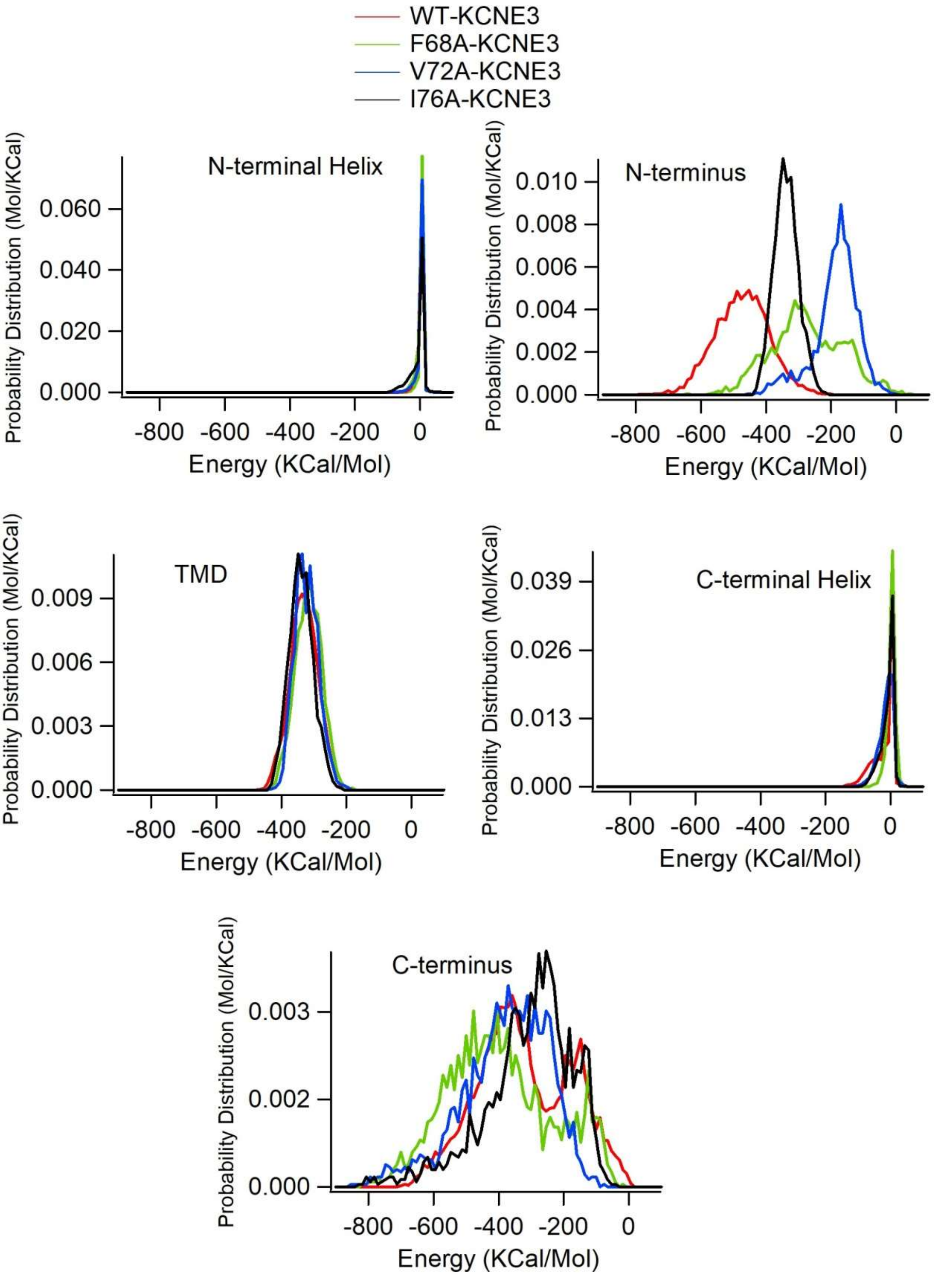

Figure S9. Histogram plots from Simulation 2 of interaction energy of KCNE3 segments with lipid bilayer membranes for WT KCNE3, F68A KCNE3, V72A KCNE3 and I76A KCNE3. The histogram plot represents probability distribution. The bin width of 9.4 was used to obtain histogram.

POPC/POPG	Average RMSD (Å) for Simulation 1			
	WT	F68A	V72A	I76A
TMD	2.0 ±0.6	0.7±0.2	1.0±0.3	0.3±0.1
N-terminal helix	0.8±0.5	0.9±0.3	0.7±0.2	2.4±0.4
N-terminus	9.4±2.0	11.8±3.6	10.9±2.1	16.7±6.0
C-terminal helix	2.2±0.7	1.4±0.5	2.2±0.8	1.8±0.8
C-terminus	3.1±0.5	4.5±1.8	13.7±4.0	6.8±1.2

POPC/POPG	Average RMSD (Å) for Simulation 2			
	WT	F68A	V72A	I76A
TMD	0.7±0.2	0.9±0.2	0.6±0.1	0.7±0.2
N-terminal helix	0.9±0.3	1.0±0.4	0.8±0.2	0.9±0.2
N-terminus	10.9±4.1	10.2±2.3	10.9±2.1	15.0±5.5
C-terminal helix	1.8±0.5	1.3±0.6	2.4±0.8	1.5±0.9
C-terminus	5.2±1.2	5.6±1.0	9.6±3.4	5.6±3.5

Table S1. Comparison of Average RMSD calculated for different sections of wild type KCNE3 and the KCNE3 in the presence of mutations (F68A, V72A, I76A) from the RMSDs shown in Figure S1 and S2 for Simulation 1 and Simulation 2. The error represents standard deviation.

POPC/POPG	Bending Angle (^{0})			
	**WT**	**F68A**	**V72A**	**I76A**
**TMD helix Simulation 1**	178.3 ±0.3	179±0.2	178.3±0.2	178.8±0.1
**TMD helix Simulation 2**	178.1 ±0.1	179±0.1	178.3±0.2	178.8±0.1

Table S2. Comparison of the average bending angles calculated from the bending angles shown in Figure S2. The error represents standard deviation.

	**Average Z-distance (Å) Simulation 1**			
	**WT**	**F68A**	**V72A**	**I76A**
**N-terminal** helix	34.5 ±3.2	46.4±3.8	43.4±2.6	41.9±3.3
**S57**	15.1±3.1	20.0±2.8	22.8±2.5	21.2±2.5
**A69**	-0.7±2.0	4.4±2.6	5.2±2.2	6.5±3.3
**S82**	-16.6±3.5	-12.7±2.5	-11.8±3.0	-10.7±3.6
**C-terminal** helix	-30.6±3.4	-31.3±6.2	-28.1±3.2	-23.9±4.7

POPC/POPG	Average Z-distance (Å) Simulation 2			
	**WT**	**F68A**	**V72A**	**I76A**
**N-terminal Helix**	34.2 ±3.1	41.9±2.8	39.2±4.7	45.8±5.3
**S57**	18.7±2.5	18.5±2.9	21.8±3.0	20.6±3.7
**A69**	1.3±2.0	3.4±2.1	6.0±2.8	5.1±3.1
**S82**	-16.9±2.1	-9.4±3.4	-11.3±3.3	-10..7±5.8
**C-terminal helix**	-28.9±7.7	-27.9±3.5	-27.5±2.5	-27.4±4.5

Table S3. Comparison of Average Z-distance calculated from the Z-distance from Simulation 1 and Simulation 2. The error is reported as the standard deviation.

	Average Interaction Energy (kcal/mol) Simulation 1			
	**WT**	**F68A**	**V72A**	**I76A**
**TMD**	-309.9 ±37.3	-323.6±39.8	-352.6±36.7	-321.8±41.1
**N-terminal helix**	-9.2±26.6	-1.2±5.1	-1.1±4.2	-1.7±7.2
**N-terminus**	-500.9±100.2	-371.1±90.0	-300.9±132.2	-355.9±103.1
**C-terminal helix**	-13.3±16.4	-1.8±12.5	-5.9±23.7	-2.2±13.3
**C-terminus**	-225.8±97.6	-266.1±151.5	-403.9±159.2	-275±146.8

	Average Interaction Energy (kcal/mol) Simulation 2			
	**WT**	**F68A**	**V72A**	**I76A**
**TMD**	-336.3 ±42.4	-322.5±40.4	-327.9±36.2	-344.0±36.7
**N-terminal helix**	-4.3±8.7	-5.5±11.8	-3.7±10.2	-11.9±20.4
**N-terminus**	-480.2±82.4	-279.5±109.7	-191.2±67.9	-344.0±36.7
**C-terminal helix**	-24.0±31.8	-3.9±12.1	-20.5±21.8	-15.1±19.5
**C-terminus**	-325.8±142.9	-401.2±161.4	385.5±133.4	-313.9±135.3

Table S4. Comparison of Average interaction energy calculated from the interaction energy from Simulation 1 and Simulation 2. The error represents standard deviation.

MA1. WT_PCA1

MA2.WT_PCA2

MB1. F68A_PCA1

MB2. F68A_PCA2

MC1. V72A_PCA1

MC2. V72A_PCA2

MD1. I76A_PCA1

MD2. I76A_PCA2

Movie M1. Movie of the fluctuations mapped onto the residues of KCNE3 TMD structure for the PC1 and PC2 for WT KCNE3 **(MA1, MA2)**, F68A KCNE3 **(MB1, MB2)**, V72A KCNE3 **(MC1, MC2),** and I76A KCNE3 **(MD1, MD2).** These movies are prepared from the Simulation 1 PCA data.
